# Active osseointegration in an *ex vivo* porcine bone model

**DOI:** 10.3389/fbioe.2024.1360669

**Published:** 2024-03-22

**Authors:** Thomas A. G. Hall, Konstantinos Theodoridis, Nupur Kohli, Frederic Cegla, Richard J. van Arkel

**Affiliations:** ^1^ Biomechanics Group, Department of Mechanical Engineering, Imperial College London, London, United Kingdom; ^2^ Non-Destructive Evaluation Group, Department of Mechanical Engineering, Imperial College London, London, United Kingdom

**Keywords:** osseointegration, osteogenesis, bioelectronics, mechanobiology, bioreactor

## Abstract

Achieving osseointegration is a fundamental requirement for many orthopaedic, oral, and craniofacial implants. Osseointegration typically takes three to 6 months, during which time implants are at risk of loosening. The aim of this study was to investigate whether osseointegration could be actively enhanced by delivering controllable electromechanical stimuli to the periprosthetic bone. First, the osteoconductivity of the implant surface was confirmed using an *in vitro* culture with murine preosteoblasts. The effects of active treatment on osseointegration were then investigated in a 21-day *ex vivo* model with freshly harvested cancellous bone cylinders (n = 24; Ø10 mm × 5 mm) from distal porcine femora, with comparisons to specimens treated by a distant ultrasound source and static controls. Cell viability, proliferation and distribution was evident throughout culture. Superior ongrowth of tissue onto the titanium discs during culture was observed in the actively stimulated specimens, with evidence of ten-times increased mineralisation after 7 and 14 days of culture (*p* < 0.05) and 2.5 times increased expression of osteopontin (*p* < 0.005), an adhesive protein, at 21 days. Moreover, histological analyses revealed increased bone remodelling at the implant-bone interface in the actively stimulated specimens compared to the passive controls. Active osseointegration is an exciting new approach for accelerating bone growth into and around implants.

## 1 Introduction

Osseointegration is the stable anchorage of a load-carrying implant by direct structural and functional connection to living bone (Shah et al., 2019). Osseointegrative implants are used across orthopaedic, oral (Buser et al., 2017), and craniofacial surgery (Granstrom, 2007) to treat millions of patients with severely disabling degenerative conditions, traumatic injuries, and congenital disorders. Initial stability of an osseointegrative implant is established by interference fit to the host bone (Immel et al., 2023). The secondary biological process wherein the bone adapts to establish long-term stability is complex (Trindade et al., 2016), typically lasting three to 6 months, with success dependent on patient metabolism (Jamsen et al., 2014), the properties of the implant biomaterial (Kaur and Singh, 2019), and the stability of initial fixation (Kohli et al., 2021).

Researchers are actively researching adjunct therapies to enhance osseointegration, with recent work exploring biophysical (Pettersen et al., 2022) and pharmaceutical approaches (Geng et al., 2020; Meng et al., 2022). Beneficial effects of electrical stimulation on the osteogenic processes that lead to osseointegration (e.g., osteoblast proliferation and differentiation) have been reported from simplified *in vitro* models, but parameters have not been optimised and the effects have not been conclusively replicated in humans (Pettersen et al., 2021; Nicksic et al., 2022). The osteogenic/osseointegrative effects of ultrasonic stimulation have also been explored in both *in vitro* cell models (Wang et al., 2014; Nagasaki et al., 2015; Wu et al., 2015; Cao et al., 2017; Feng et al., 2019; Ambattu et al., 2022) and *in vivo* animal models (Iwai et al., 2007; Ustun et al., 2008; Hsu et al., 2011; Liu et al., 2012; Zhou et al., 2016; Ruppert et al., 2019). As is the case for electrical stimulation, the effects of low-intensity pulsed ultrasound are not well understood, have not been optimised, and have not been replicated in humans (McCarthy and Camci-Unal, 2021). The delivery of stimulation from an external source is a plausible reason for the mixed results, as osteocytes, the most mechanosensitive bone cells (Bonewald, 2011), are embedded within mineralised tissue which reflects ultrasound. Any evaluation of active osseointegration must therefore consider the effect of bone structure on the stimulus in addition to the response of heterogeneous cell types.

*Ex vivo* bioreactor models provide an alternative to live animal testing for early development of implant fixation technology for applications where the interplay between cellular response and bone structure is important (Dua et al., 2021; Zankovic et al., 2021; Hall et al., 2023; Kohli et al., 2023). The model is an intermediate between *in vitro* studies of isolated biological processes and *in vivo* studies with many potentially confounding effects: it enables study under tightly controlled near-physiological conditions whilst preserving the inherent cellular diversity and extracellular structure of natural bone. Thus, the aim of this study was to investigate the effects of active stimulation on osseointegration in an *ex vivo* bone bioreactor model. First, the osseoconductivity of a rough titanium implant surface was evaluated with osteoblast-like cells. The effects of active treatment, delivered via the implant surface, on ongrowth from living porcine bone explants were then investigated, with comparisons to ultrasonic treatment from a distant source and passive controls.

## 2 Materials and methods

### 2.1 Implant manufacture

Twenty-one passive cylindrical implants (Ø10 mm × 3 mm) were additively manufactured from commercially pure titanium powder (ASTM B348 Grade two spherical powder, Ø15-45 μm, Carpenter Additive, United States) using a powder bed fusion system (AM250, Renishaw, UK). Surface roughness was created on the bone-facing base of the implants by adding randomized sinusoidal-based contours to the part, as previously described (van Arkel et al., 2018), to yield roughness of Rz = 730 μm, Ra = 75 μm, Rq = 100 μm; this level of surface roughness is similar to that of contemporary cementless joint replacement implants.

Six of the passive implants were modified to deliver an active osseointegration stimulus. It has previously been shown that embedded ultrasound transducers are effective at diagnostically monitoring small changes at the implant/bone interface (Hall et al., 2021). Inspired by those findings, a system that delivers electrical power to an implant and stimulates tissue at the implant-bone interface via an embedded ultrasound transducer was designed. Thickness-mode piezoelectric transducers (PI Ceramic PRYY+0442; Ø10 mm × 1 mm) were embedded within a biocompatible polymer insert (Stratasys ABS-M30i) and bonded to the titanium discs using a thin layer of epoxy (Loctite Double-Bubble). Electrical contact was established to the positive electrode by soldering and to the negative electrode with a biocompatible Steel 316L grub screw. PDMS was used to encapsulate the circuitry and provide a 3-mm thick backing for the transducer.

### 2.2 *In Vitro* osseoconductivity culture

**FIGURE 1:**
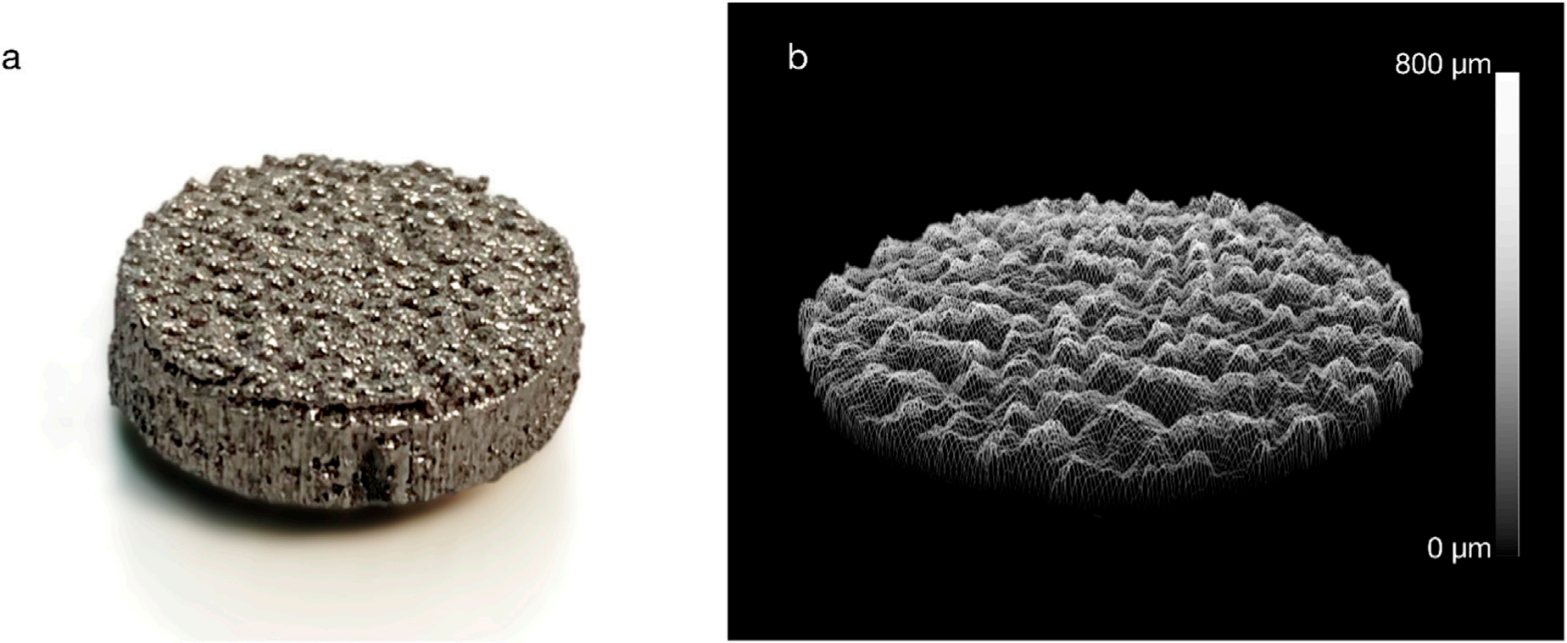
**(A)** An example additive manufactured titanium implant and **(B)** a surface roughness measurement (Ra = 75 µm).

Murine preosteoblasts from the MC3T3-E1 cell line (ATCC CRL-2593) were proliferated for three passages in cell culture medium: α-MEM (ThermoFisher 11095080) supplemented with 10% foetal bovine serum (ATCC 30-2020) and 1% penicillin-streptomycin (ThermoFisher 15070063). At 75% confluency on the final passage, the cells were trypsinised and seeded directly onto the rough surface of the additive-manufactured titanium discs (Figure 1; n = 8) at a density of 5,000 cells/cm^2^. Specimens were cultured in twelve-well plates for 7, 14, 21, and 28 days (n = 2 per time point) in normoxic conditions at 37°C and 5% CO_2_. Cell culture medium was replaced every 7 days.

### 2.3 *Ex Vivo* active osseointegration culture

**FIGURE 2:**
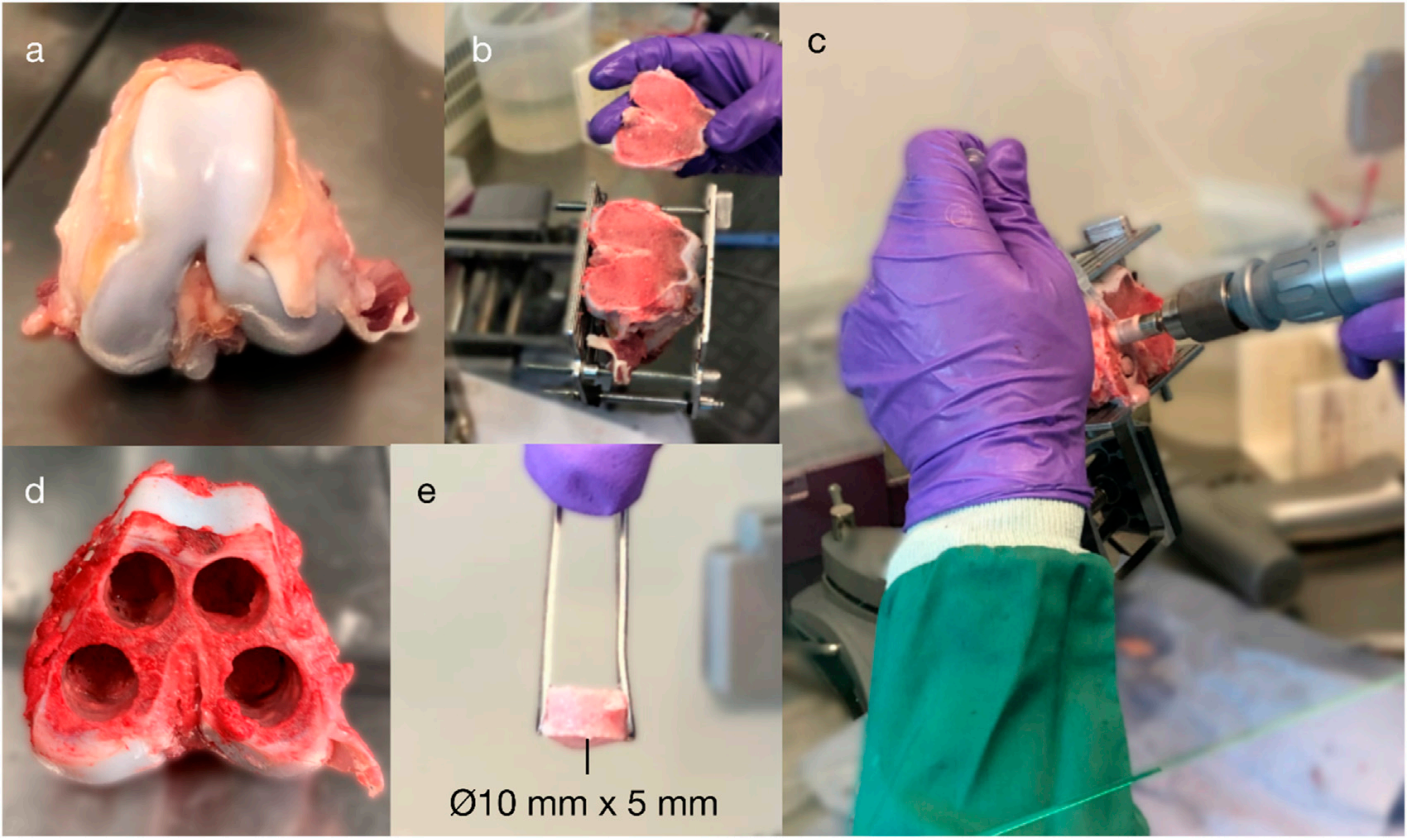
Explantation of porcine cancellous bone from the distal femoral condyles: **(A)** isolation of the distal femur; **(B)** exposure of the subchondral bone; **(C–D)** extraction of four cancellous bone cores (⌀10 mm) from the medial and lateral and femoral condyles (up to 25-mm length); and **(E)** a core after being transected to a 5-mm length.

Research was registered as part of the PI’s annual declaration in accordance with the host institution’s policy for research with animal tissue samples. Fresh trabecular bone cylinders (Ø10 mm × 5 mm; n = 24) were prepared from distal porcine femora (age: 18 weeks; weight: ∼75 kg); the donor animals had already been euthanised for unrelated research that did not affect bone metabolism or healing. Specimens were transected at the mid-femur and mid-tibia, exsected to the level of the stifle (‘knee’) joint capsule, and immersed in ethanol for 7 minutes before being transferred to a laminar flow hood. Cancellous bone cylinders were then extracted from the distal femoral condyles in a sterile manner (Figure 2): power tools, attachments, and clamps were autoclaved; the bioreactor was immersed in 70% ethanol after assembly and irradiated with ultraviolet (UV) light for 30 min on each side; and all other equipment was wiped with 70% ethanol and irradiated with UV.

Specimens were extracted using an oscillating saw blade and a diamond holesaw; ice-cold Dulbecco’s phosphate-buffered saline (DPBS; ThermoFisher 14190250) with 1% penicillin/streptomycin (ThermoFisher 15070063) was used to minimise thermal necrosis whilst cutting with low rotational speed. Specimens were washed in DPBS and vortexed for 30 s to reduce cutting debris. Within 5 hours of euthanisation, all bone cores were prepared and immersed in cell culture medium, which was composed of DMEM/F-12 with HEPES (ThermoFisher 11330057) plus 10% foetal bovine serum (ThermoFisher 10500064) and 1% penicillin/streptomycin (ThermoFisher 15070063). During culture, bone cores were incubated in normoxic conditions at 37°C and 5% CO_2_.

**FIGURE 3:**
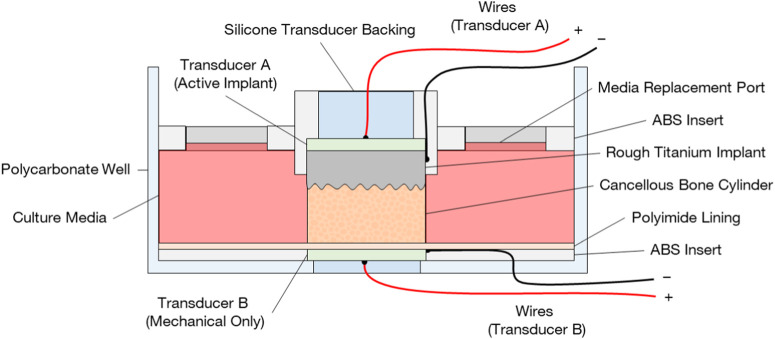
Bioreactor design for *ex vivo* evaluation of active osseointegration of cancellous bone into rough additive-manufactured titanium implant surface with two different treatment conditions: **(A)** active osseointegrative implant delivering direct stimulation (combined electromechanical) through the implant and **(B)** a distant ultrasound source delivering low-intensity pulsed ultrasound through the bone. Note in configuration **(A)**, the transducer **(B)** was not present, and *vice versa*.

The bone samples were cultured in a custom-built bioreactor (Figure 3) and divided between four conditions: A: the active osseointegrative implants (n = 6); B: passive implants treated with low-intensity pulsed ultrasonic delivered through the bone (n = 6); C: passive implant controls with no stimulation (n = 9); and D: bone-only controls (n = 3). For Condition B, the same transducers were used as in Condition A. The transducers were embedded into the base of the bioreactor using a thin layer of epoxy and a biocompatible polymer insert, which electrically isolated the transducers from the bone core.

In both treatment conditions (A and B), bone cores were stimulated daily for 30 min. The ultrasound stimulus replicated clinical low-intensity pulsed ultrasound systems (frequency 2 MHz, power density 30 mW/cm^2^) (Harrison and Alt, 2021). Control specimens were acoustically isolated from treated specimens. Treatment began after 24 h of acclimatisation to the *ex vivo* environment and lasted 21 days, at which point the culture was terminated. Fresh media (8 mL) was supplied to the specimens on the days 0, eight and 15, and retrieved on days 7, 14 and 21. The media on days 7–8 and days 14–15 contained fluorescent dyes for mineral labelling and was replaced after 24 h.

### 2.4 Viability staining

A two-colour assay (ThermoFisher L3224; Calcein-AM/Ethidium homodimer) was used to stain live/dead cells to determine cell viability, proliferation and distribution on the titanium implant surfaces: (i) after 7, 14, and 28 days of *in vitro* culture (n = 1 per timepoint) and (ii) after 21 days of *ex vivo* culture (n = 1 per condition). Specimens were incubated with the stains for 2 hours and washed three times with phosphate-buffered saline to remove the excess of residual dye. Confocal images (Leica Microsystems TCS SP5) were acquired from all specimens; widefield microscopy images (Zeiss Axio Observer) were acquired from *ex vivo* discs to determine ongrowth at the macroscopic level.

### 2.5 Mineralisation staining

Fluorophores (Calcein, Sigma-Aldrich C0875; Alizarin Red S, Sigma-Aldrich A5533) were administered on the seventh and 14th days of stimulation through the media (50 μg/mL) for 24 h. Calcein (excitation: 470 nm; emission: 509 nm) and Alizarin Red S (excitation: 532 nm; emission: 620 nm) bind to the calcium ions in growing calcium phosphate/carbonate crystals, forming fluorescent complexes that label newly mineralised bone at each timepoint (van Gaalen et al., 2010). Post-incubation, all titanium discs were imaged using widefield microscopy (Zeiss Axio Observer). Mean fluorescent intensity on the disc for each stain was recorded and background fluorescence subtracted.

### 2.6 Scanning electron microscopy

Titanium discs (n = 2 per *in vitro* timepoint; n = 3 per *ex vivo* condition) were fixed in 2.5% glutaraldehyde with 0.1 M phosphate buffer (pH 7.4) overnight, dehydrated through a series of ethanol solutions (20%-100%; 7 min per solution), and air-dried for analysis. The discs were then sputter-coated with chromium (thickness: 15 nm) and imaged using a scanning electron microscope (TESCAN MIRA) for signs of cellular ongrowth.

### 2.7 Calcium concentration

Supernatants from each timepoint in the *ex vivo* culture were retrieved and analysed for calcium concentration using a colorimetric assay (Sigma-Aldrich MAK022; n = 3 for each condition). The chromogenic reagent (o-Cresolphthalein complexone) forms a violet-coloured complex (absorbance: 575 nm) with Calcium ions (Ca^2+^) in alkaline solution. Absorbance readings were taken in triplicate for each sample, fresh media, and blank media without any reagent, as per manufacturer’s instructions.

### 2.8 Alkaline phosphatase

The retrieved media from the *ex vivo* culture was analysed for alkaline phosphatase activity (ALP) using a fluorometric assay (Sigma-Aldrich MAK411; excitation: 360 nm; emission: 440 nm). ALP is a major regulator of osteoblast differentiation and bone extracellular matrix mineralisation, with a vital role in the supply of inorganic phosphate ions for the formation of hydroxyapatite, the primary mineral component of bone (Blair et al., 2017).

### 2.9 Media pH

Cell culture media (ThermoFisher 11330057) was supplemented with phenol red–a non-toxic pH indicator that is colour-sensitive in the range 6.8–8.2 (yellow to fuchsia). The colour of the media was monitored during the culture to ensure near-physiological pH (∼7.4) was maintained. Secondary measures of pH were acquired directly from the media upon retrieval using indicator strips (VWR 315082P).

### 2.10 Histology

Bone specimens were fixed in 10% neutral buffered formalin overnight at room temperature, washed in 0.1 M phosphate-buffered saline (pH 7.4), and decalcified over 21 days in an acid solution (8% EDTA; 0.8% NaOH) with daily changes. After decalcification, specimens were washed with tap water, dehydrated through a graded series of ascending alcohol solutions and toluene, and embedded in paraffin wax. Sections perpendicular to the bone-implant interface were cut from the centre of each specimen using a rotary microtome (Leica Biosystems RM2235) to a thickness of 7 µm and mounted on microscope slides. Slides were deparaffinised, rehydrated, and stained with Haematoxylin and Eosin (H&E; BioGnost HE-RTU-100T), Masson-Goldner’s trichrome (BioGnost MGT-100T), Movat’s Pentachrome (BioGnost MOV-100T), and Picrosirius Red (Abcam ab150681), according to manufacturer protocols.

### 2.11 Immunohistochemistry

Decalcified sections were deparaffinised in Citroclear (Genta Medical CIT050; 2 × 15 min) and taken through secondary rinses in 99% industrial methylated spirit (Solmedia; IMS005; 2 × 30 min). Endogenous peroxidases were blocked by incubating sections in 1% hydrogen peroxide in absolute methanol for 10 min, and sections were then rinsed in cold-running tap water for at least 10 min. Antigen retrieval was carried out by incubating sections in citrate buffer (pH 6) overnight: sections were put into the buffer whilst still cold and were allowed to warm gradually. The following day, sections were rinsed in PBS and a PAP pen ring was drawn around the sample. Sections were blocked by incubating in 5% normal goat serum (Sigma N502L) in PBS for 1 h at room temperature; excess serum was tapped off; and sections were rinsed in PBS (2 × 2 min). Endogenous biotin, biotin receptors, and avidin binding sites were blocked with an Avidin/Biotin Blocking Kit (2BScientific SP-2001), which was used in accordance with the manufacturer’s instructions, and sections were again rinsed in PBS (2 × 2 min). Positive sections were incubated overnight with polyclonal rabbit anti-osteopontin primary antibody (Abcam ab231736; 1.25 μg/mL) in 5% goat serum; isotypic controls were incubated in Rabbit IgG at the same concentrations; and PBS controls were maintained in PBS overnight at 4°C. All sections were rinsed the following day in wash buffer solution (pH 7.4; 3 × 5 min), which was composed of 1% Triton X-100 (Millipore 648643) in PBS. Sections were then incubated in biotinylated goat anti-rabbit secondary antibody (Jackson ImmunoResearch 111–066-046) at 1:250 PBS dilution for 1 h at room temperature, followed by rinses in the wash buffer (3 × 5 min). The VectaStain ABC kit (2BScientific PK-4000) was then applied to all sections according to the manufacturer’s instructions, and sections were again rinsed in wash buffer (3 × 5 min). Sections were incubated in DAB substrate (2BScientific SK-4100; 2-10 min per sample) with microscopic control: the reaction was stopped with cold-running tap water when the target antigen was crisp brown and the background was clear. Nuclei were counterstained with Harris Haematoxylin for 20 s and blued in running tap water. Finally, sections were dehydrated, cleared, and mounted.

Slides were analysed quantitatively for osteopontin (OPN) expression: all OPN-positive and OPN-negative osteocytes within 500 µm of the implant-bone interface were counted. Osteopontin expression was reported as a percentage of total osteocytes. Osteopontin is an adhesive extracellular matrix protein with a prominent role in early osseointegration: it is secreted onto the damaged trabecular and implant surfaces (McKee et al., 2011) and becomes mineralised as ‘cement lines’ whilst devoid of collagen fibres. *De novo* bone formation proceeds from these cement lines on both the damaged trabeculae (distance osteogenesis) and the implant itself (contact osteogenesis).

## 3 Results

### 3.1 Cultivation

**FIGURE 4:**
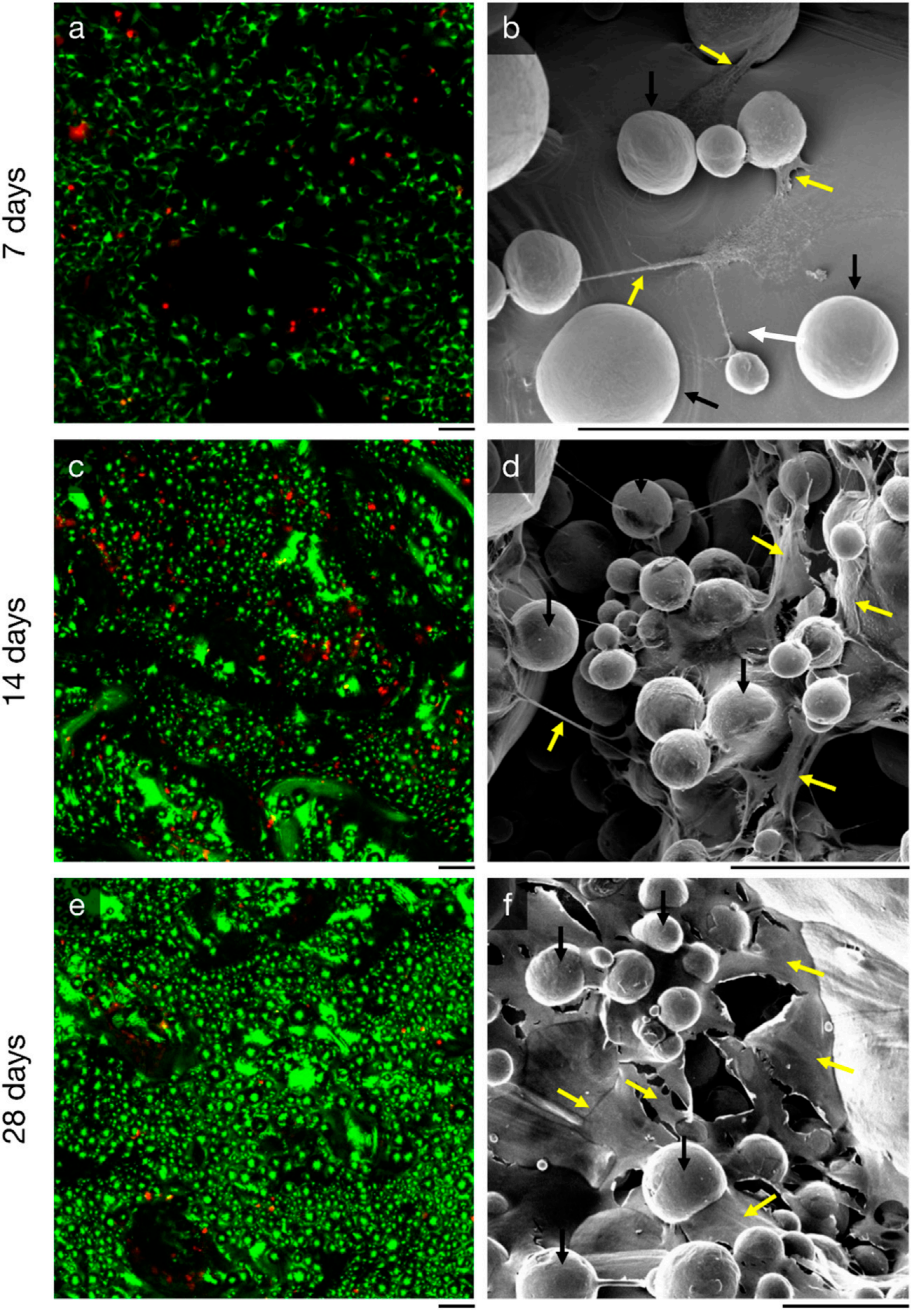
Proliferation of MC3T3-E1 murine preosteoblasts on additive-manufactured rough titanium surface (Ra = 75 µm) observed by (left, **(A, C, E)** live-dead staining and (right, **(B, E, F)** scanning electron microscopy with energy 10 keV and beam current 300 pA. Three time points are shown: (top, **(A, B)** at 7 days, (middle, **(C, D)** 14 days, and (bottom, **(E, F)** 28 days. The black line directly beneath each image is scaled to 100 µm. The spherical features (black arrows) in the scanning electron microscopy images are semi-fused titanium power particles, something that is common for all additive manufactured parts. Cells and extracellular matrix (yellow arrows) can be seen between these spherical titanium features; their structures are fibre/cord-like after 7 days, and more sheet/surface-like after 21 days.

Cell-seeded specimens were cultured *in vitro* for 28 days; high viability (>95%; Figure 4) was maintained in all samples extracted on days 7, 14, and 28. Few dead cells were observed. Additionally, uniform cell proliferation and cell distribution was observed on all timepoints during the culture.

**FIGURE 5:**
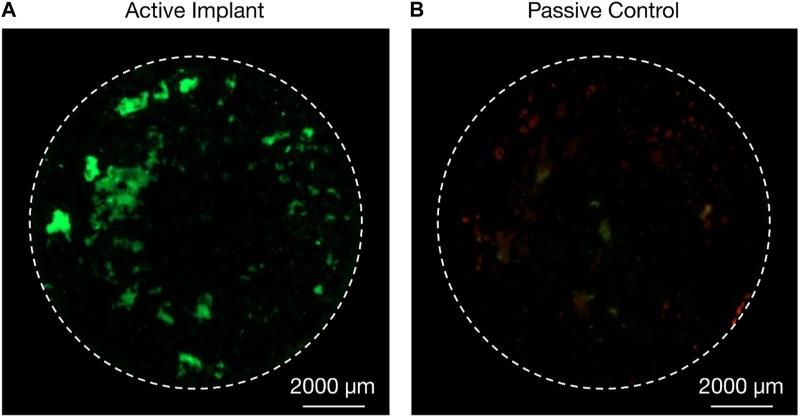
Live/dead (green/red) staining of cells from ongrowing tissue derived from porcine femoral condyle explant onto additive-manufactured rough titanium discs after 22 days in an *ex vivo* bioreactor; **(A)** higher proliferation of cells and higher viability was observed on the active osseointegrative implant (treatment A) after 21 days of treatment compared to **(B)** passive implant controls (treatment C).

*Ex vivo* specimens (n = 24) were successfully explanted from the medial and lateral porcine femoral condyles; 22 specimens were cultured for 22 days and used for analyses; two cultures (1 C and one D specimen) were terminated on the 10th day due to contamination and were not included in the analyses. Ongrown tissue of high viability was observed on the titanium discs of stimulated specimens (Figure 5A, B), whilst ongrown tissue of less expanse and less viability was observed on the titanium discs of the passive controls (Figure 5B, C). All active osseointegrative implant specimens (A) and all-but-one distant ultrasound specimens (B) had a weak degree of adhesion–enough for the bone to adhere to the implant under its own weight - at the implant-bone interface when the culture was terminated; no adhesion was observed in any of the passive controls (C). The media of active osseointegrative implant specimens was lighter upon retrieval (peach-coloured; pH ∼7.1) than distant ultrasound specimens and passive controls (red-coloured; pH ∼7.4); pH values were confirmed using indicator strips.

### 3.2 Osteoconductivity

MC3T3-E1 murine preosteoblasts had proliferated over the entire rough titanium surface within 7 days (Figure 4A); the preosteoblasts had flattened on the surface with filopodia and there was early evidence of organic matrix deposition (Figure 4B). By 14 days, cells had continued to proliferate (Figure 4C) and organic structures spanning up to 200 µm between semi-sintered particles were observed (Figure 4D). High density cell clusters were observed after 28 days of *in vitro* culture (Fig, 4e), indicating osteoblast maturation, which further resulted in more expansive new extracellular matrix formation than previous timepoints (Figure 4F).

### 3.3 Bone ongrowth

**FIGURE 6:**
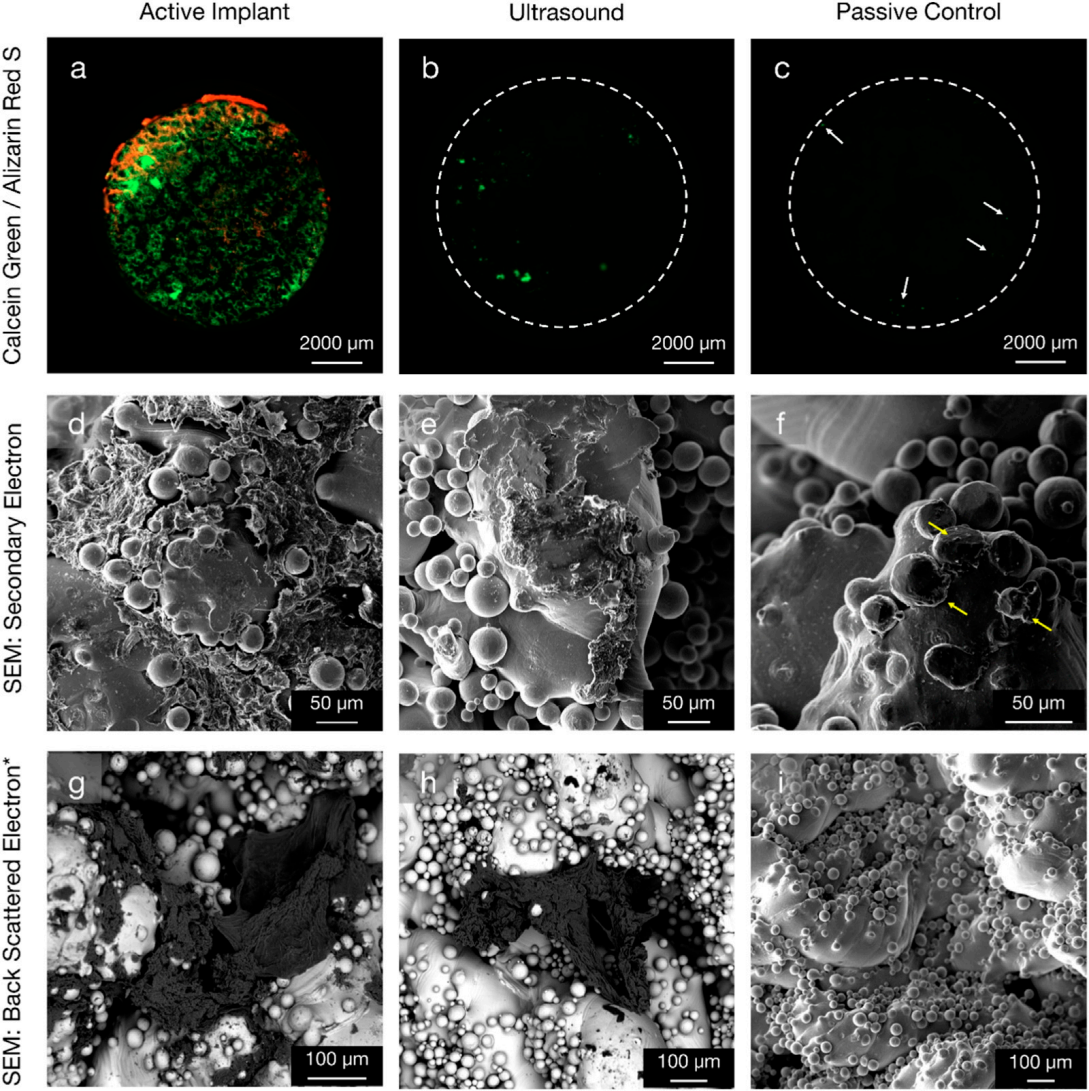
Comparison of new bone formation on the titanium implant in three configurations: (left column, **(A, D, G)** active osseointegrative implant; (middle column, **(B, E, H)** low-intensity pulsed distant ultrasound stimulation through bone; and (right column, **(C, F, I)** passive implant control with no stimulation. (Top row) widefield fluorescent microscopy provides evidence of enhanced mineralisation on the seventh (green) and 14th (red) days of culture for active implant specimens compared to the ultrasound stimulation and passive control specimens. Note day-7 mineralisation is present even for the passive control (white arrows). (Middle row) secondary electron SEM and (bottom row*) back-scattered electron SEM with energy 10 keV and beam current 3 nA at the 3-week endpoint. Widespread, surface-like coverage of extracellular matrix was observed on the surface of active implant specimens. The morphology of this tissue formation was like that observed in the pre-osteoblast cell seeding experiment (Figure 4). Smaller depositions were also visible on the surface of distant ultrasound specimens. Very limited deposition (yellow arrow) was observed on the passive controls. *Figure **(I)** is Secondary Electron SEM.

**FIGURE 7:**
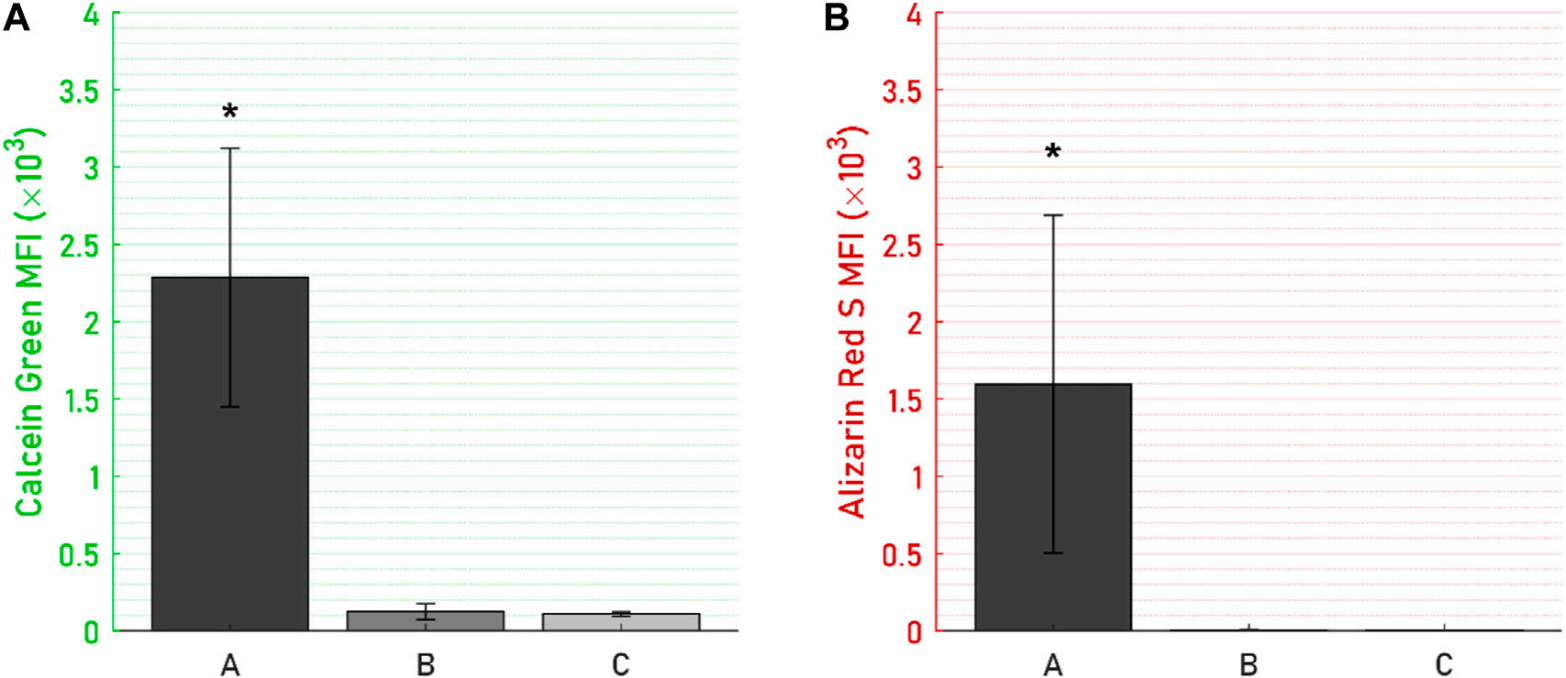
Mean fluorescent intensity (MFI) on the titanium disc for **(A)** Calcein Green, day-7 mineralisation stain, and **(B)** Alizarin Red S, day-14 mineralisation stain, were significantly higher for **(A)** the active osseointegrative implant *versus* **(B)** passive implant with distant ultrasound stimulation and **(C)** passive control. Asterisk (*) indicates significant difference (*p* < 0.05) relative to the passive implant control.

**FIGURE 8:**
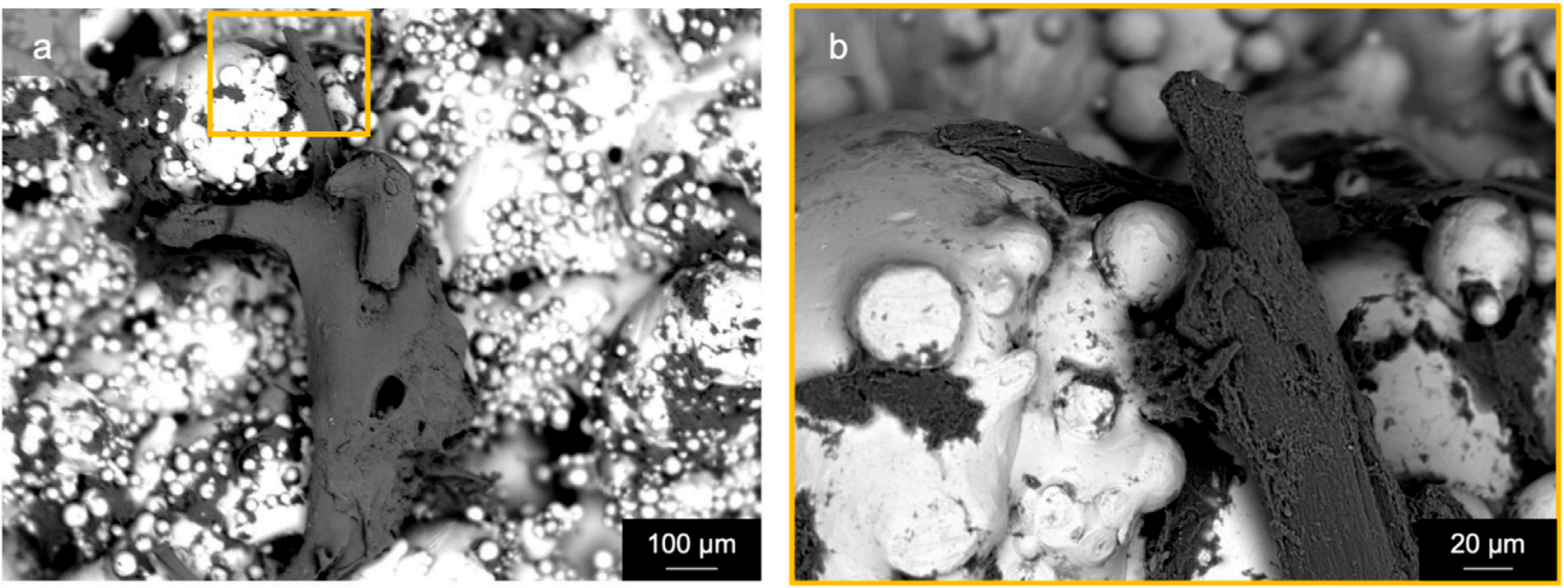
Scanning electron microscopy (back-scattered electron) revealed a mature bone fragment (length: ∼1 mm) on an active osseointegrative implant surface that had begun to integrate with the semi-sintered particles on the rough titanium implant surface: **(A)** macroscopic view and **(B)** microscopic detail.

Specimens from the active osseointegrative implant (WFM; Figure 6A) exhibited mineralisation across almost the entire surface on the seventh day of culture (Calcein Green) with continued mineralisation by the 14th day (Alizarin Red S). Mineralisation at both time points was more than ten times higher than the passive controls (WFM; Figure 6C) as measured by mean fluorescent intensity (Figure 7; *p* < 0.05). By the end of the 3-week culture, there was near-total coverage of the titanium implant surface with extracellular matrix in active osseointegrative implant specimens (SEM; Figure 6D); continuous tissue formations had spanned distances up to 2 mm; and irregular woven bone structures had bridged between and enveloped semi-sintered particles on the implant surface (BSE; Fig, 6g). A large mature bone fragment was found integrating with one active osseointegrative implant specimen (BSE; Figure 8A); continuous bone formation emanating from the fragment had begun to integrate with the semi-sintered particles on the titanium implant surface (BSE; Figure 8B).

A lesser amount of day-seven mineralisation was observed in specimens that had been subject to distant ultrasonic stimulation and the passive controls (WFM; Figures 6B, C), dissipating to near-zero mineralisation by the 14th day. Extracellular matrix had also been deposited onto the titanium by the endpoint analysis in distant ultrasound specimens (SEM; Figure 6E); tissue formations were less expansive and appeared less mature compared to active osseointegrative implant specimens; but similar woven structures were observed (BSE; Figure 6H). Some cells and organic deposits were observed on the titanium implant surface of the static control specimens (SEM; Figure 6F), but the surface was devoid of any mature, expansive organic structures (BSE; Figure 6G).

### 3.4 Media biomarkers

**FIGURE 9:**
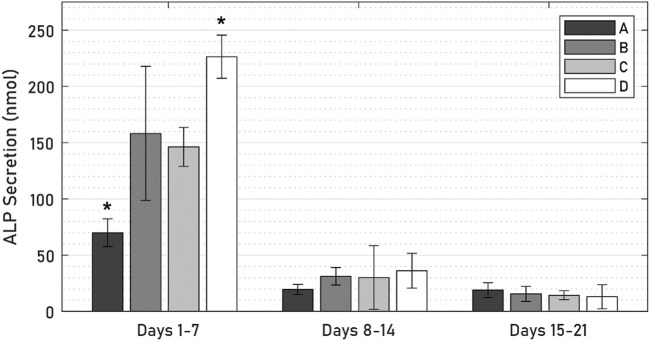
ALP levels across 21 days of culture between **(A)** active osseointegrative implant, **(B)** passive implant with distant ultrasound treatment, **(C)** passive implant control, and **(D)** bone-only control. Asterisk (*) indicates significant difference (*p* < 0.05) relative to the passive implant control.

ALP was expressed by all specimens throughout the 21-day culture period (Figure 9), though levels of ALP secretions decreased steeply from an initial peak in the first 7 days. In this initial period, ALP levels of the active osseointegrative implant specimens were 52% lower than those of the passive control specimens (*p* = 0.010); Distant ultrasound treatment had no effect on ALP secretions (*p* = 0.808); and ALP secretions without any titanium implant were increased by 55% (*p* = 0.048). There was no difference in ALP secretions beyond the initial 7-day period.

**FIGURE 10:**
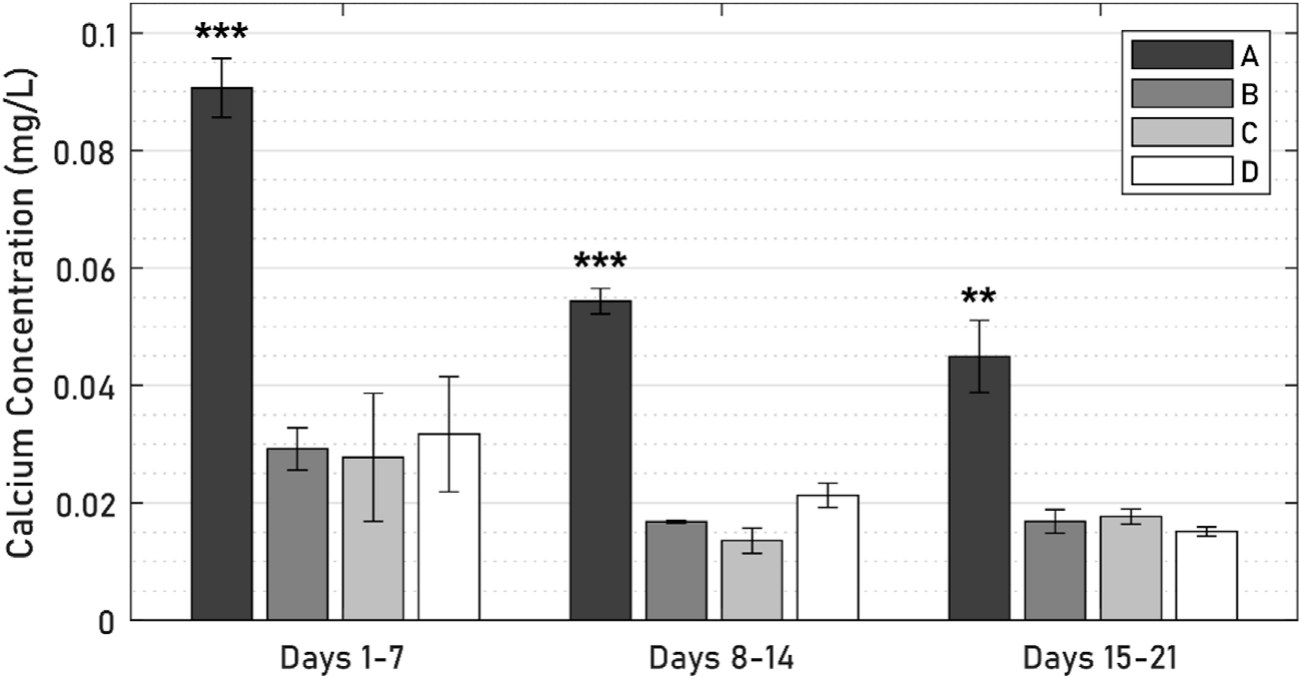
Calcium levels across 21 days of culture between **(A)** active osseointegrative implant, **(B)** passive implant with distant ultrasound treatment, **(C)** passive implant control, and **(D)** bone-only control’. Asterisks indicate significant difference (*: *p* < 0.05; **: *p* < 0.005; ***: *p* < 0.001) relative to the passive implant control.

Calcium levels in the active osseointegrative implant specimens were significantly elevated *versus* the passive control at every timepoint (Figure 10; all *p* < 0.01). Calcium levels in the active osseointegrative implant specimens did decrease throughout culture, but remained higher than all other samples, which showed constant calcium levels, without any differences detected between them.

### 3.5 Histology

**FIGURE 11:**
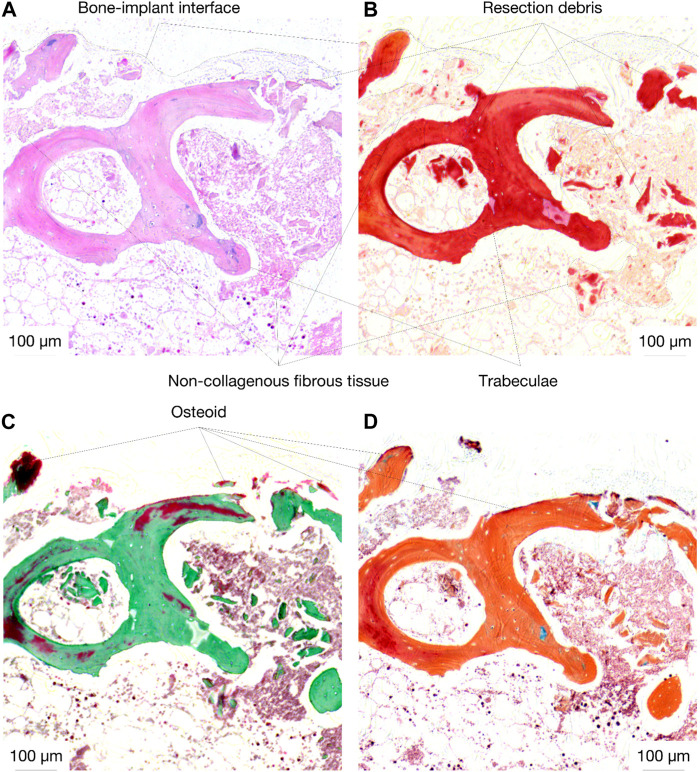
Histological staining **(A)**: H&E; **(B)** Picrosirius Red; **(C)** Masson-Goldner Trichrome; **(D)** Movat Pentachrome) of an active osseointegrative implant specimen at the bone-implant interface, showing resection debris, non-collagenous fibrous tissue formation, and osteoid seams in apposition to the interface (red in Masson-Goldner and Movat stains).

**FIGURE 12:**
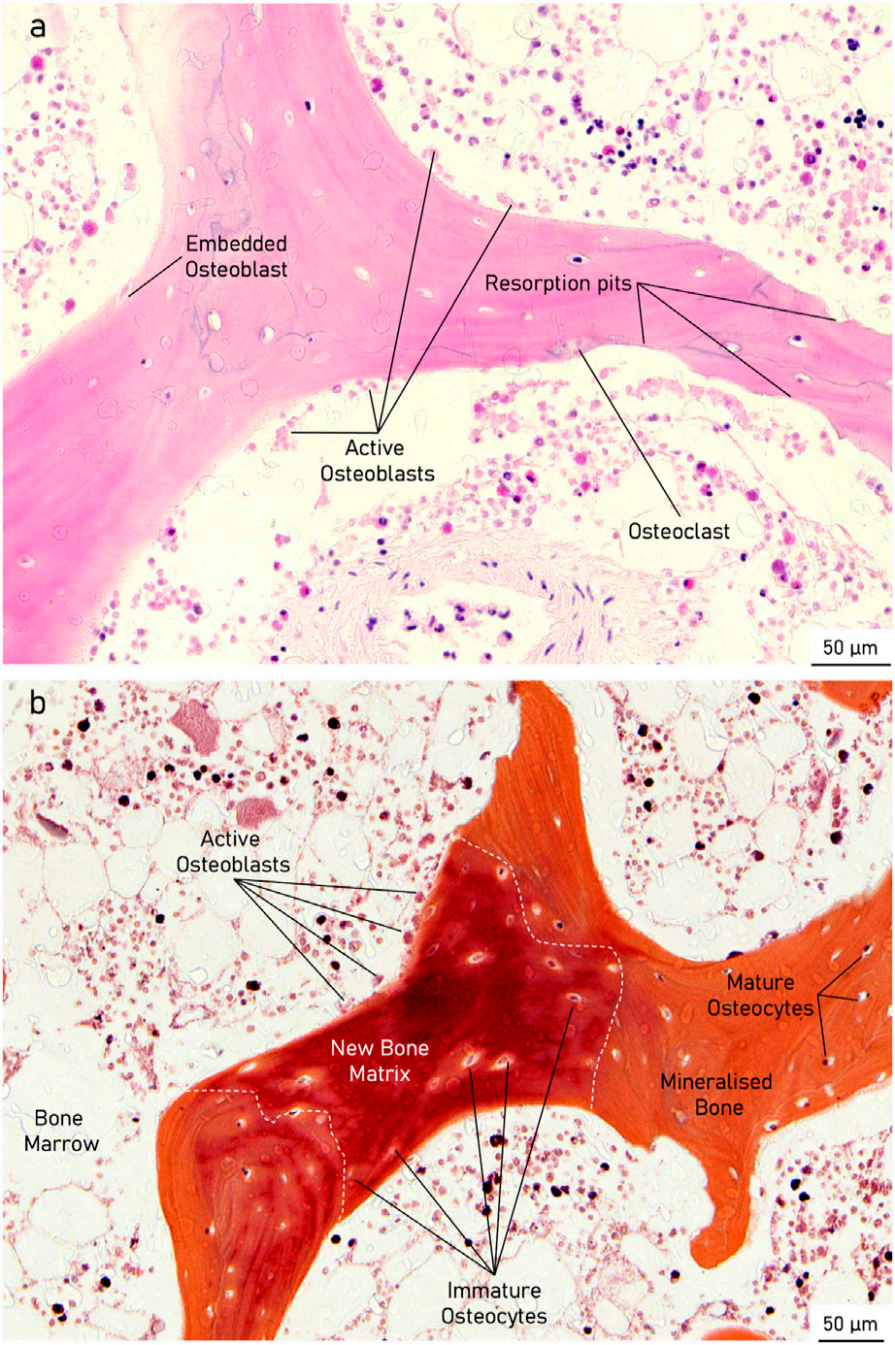
Histological staining of an active osseointegrative implant specimen showing **(A)**: H&E) a bone remodelling compartment close to the bone-implant interface with active osteoblast formation and osteoclast resorption and **(B)**: Movat Pentachrome) active osteoblasts depositing new bone matrix, becoming entrapped within their osteoid section, and maturing into osteocytes in the bulk tissue.

Histological analysis of the specimens treated with the active osseointegrative implant revealed bone fragments–cleaved from the bulk trabecular structure during the initial extraction–amidst newly-formed non-collagenous fibrous tissue (Figure 11). There was evidence of new bone matrix deposition (Figure 11; 12A) in direct apposition to the implant surface, growing from both the bulk trabecular structure and the resection debris fragments. Furthermore, bone remodelling (Figure 12: formation and resorption) was seen in the bulk tissue of the active osseointegrative implant specimens. Less osteogenic activity was observed at the surfaces open to the media and in contact with the polymer base of the bioreactor (Supplementary Figures S2-S3); similar resection debris and non-collagenous fibrous tissue formations were seen at these surfaces.

**FIGURE 13:**
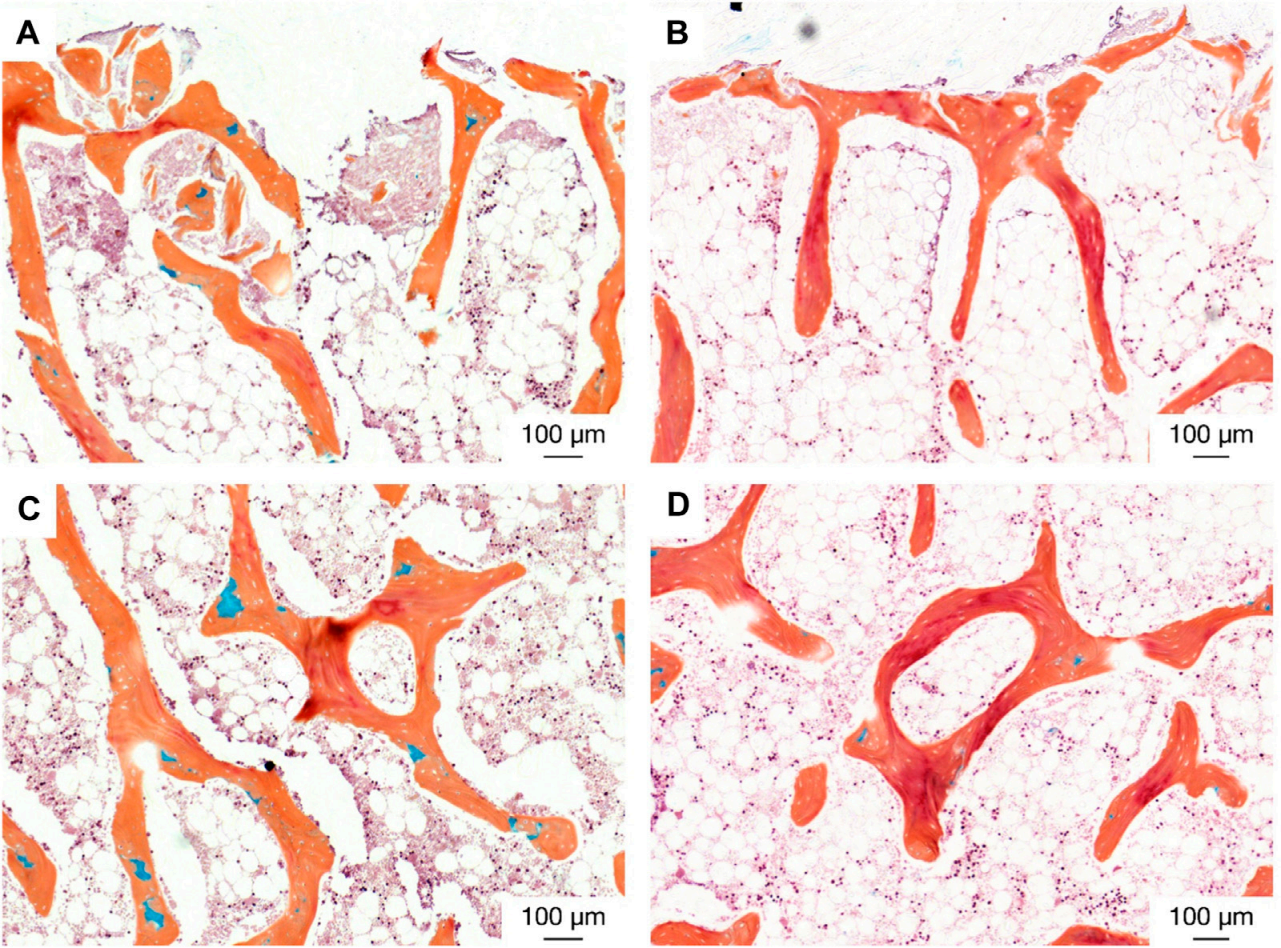
Histological staining (Movat pentachrome) indicating new bone formation (red) at (top; **(A, B)** the bone-implant interface and (middle; **(C and D)** in the bulk tissue for (left; **(A, C)** the distant ultrasound specimens and (right; **(B, D)** the passive implant controls.

There were also similar bone fragments and non-collagenous tissue formations near the interface in the distant ultrasound specimens and the passive controls, but less osteoid seams were observed in apposition to the titanium implant surface; (Figures 13A, B). Remodelling was evident in the bulk tissue, the surfaces open to the media, and the surfaces in contact with the polymer base of the bioreactor in the distant ultrasound and passive control specimens (Figures 13C, D). There was no indication of mass resorption of cancellous bone in any of the specimens.

Additional histology images are available as Supplementary Material.

### 3.6 Immunohistochemistry

**FIGURE 14:**
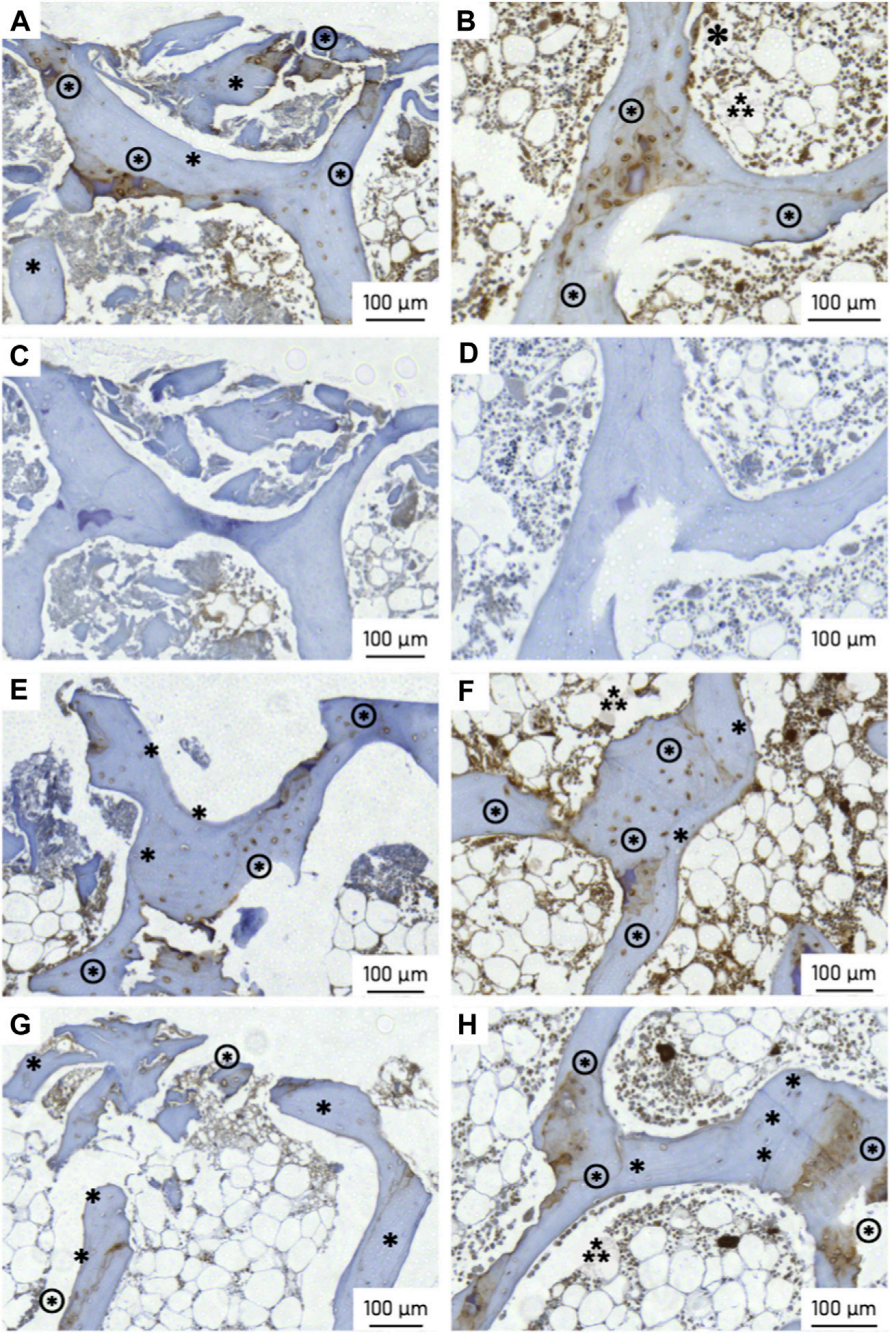
Osteopontin (OPN) expression at the (left) bone-implant interface and (right) in the bulk trabecular structure for (top; **(A, B)** an active osseointegrative implant specimen, **(C, D)** the corresponding isotypic controls for the active osseointegrative implant specimen, **(E, F)** a distant ultrasound treated specimen, and (bottom; **(G, H)** a passive implant control. Annotations indicate OPN-positive osteocytes (brown cytoplasm; ⊛), OPN-negative osteocytes (blue nuclei; ∗), osteoblasts (⁂), and osteoclasts (✽; visible in image b only).

**FIGURE 15:**
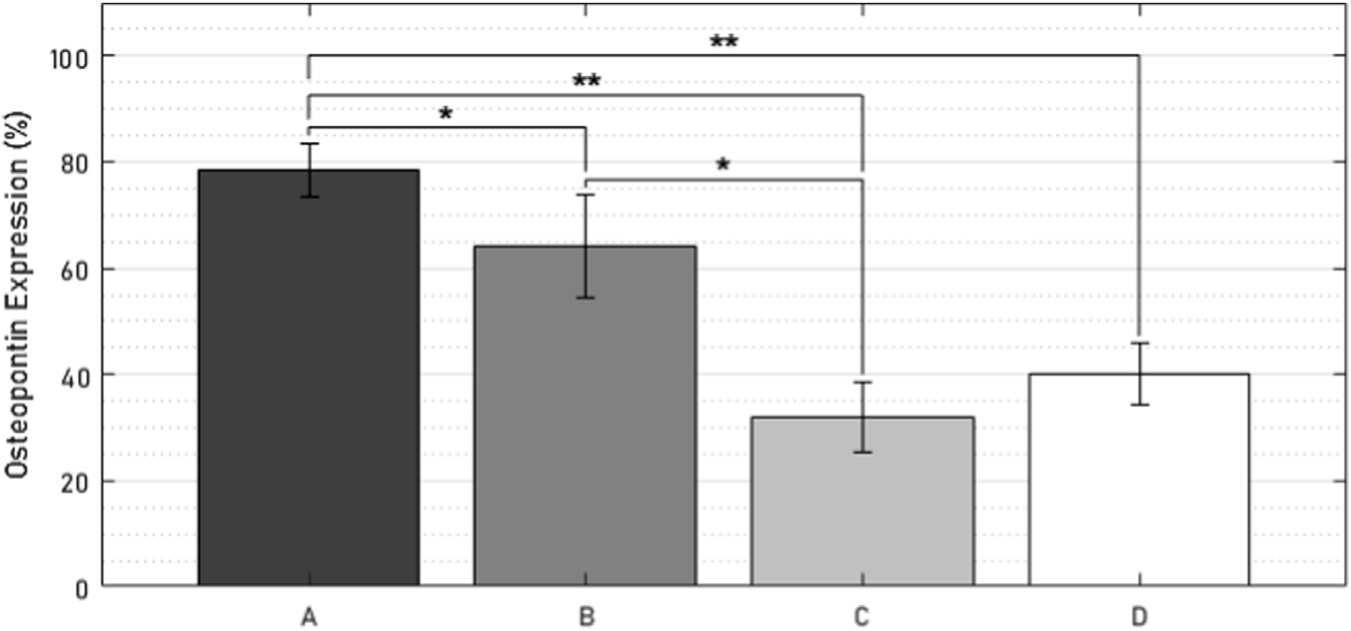
Percentage of osteopontin-positive cells within 500 µm of the bone-implant interface in **(A)** the active osseointegrative implant, **(B)** passive implant with distant ultrasound treatment, **(C)** passive implant control, and **(D)** bone-only control. Asterisks indicate significant difference (*: *p* < 0.05; **: *p* < 0.005).

Evidence of osteopontin expression was observed in all specimens (Figure 14). At the bone-implant interface, the number of osteopontin-expressing osteocytes was 47 percentage points (pp) higher in the active osseointegrative implant compared to the passive implant controls (2.5 times greater, *p* < 0.005; Figures 14A, G; Figure 15). Similarly, the distant ultrasound led to a 32 pp higher osteopontin expression compared to the passive implant controls (2 times greater, *p* < 0.05; Figures 14E,G; Figure 15). Comparing the two stimulated cases, the active osseointegrative implant led to a 15 pp higher osteopontin expression *versus* the distant ultrasound (1.2 times greater, *p* < 0.05; Figures 14A, E; Figure 15). Osteopontin was also expressed by osteocytes in the bulk of tissue of all specimens, with further expression by osteoblasts and osteoclasts in remodelling zones (Figure 14B, F, H).

## 4 Discussion

This research was the first *ex vivo* study of an active osseointegration: an intermediate between *in vitro* experiments on isolated cell types and *in vivo* trials in live animals. The model has advanced on earlier *in vitro* research by investigating the effects of active stimulation under tightly controlled physiological conditions at a tissue level, wherein the cellular diversity and extracellular structure of natural bone was inherently captured. Effects of active treatment on new bone formation on the implant (contact osteogenesis) and in the periprosthetic tissue (distance osteogenesis) were referenced against treatment with stimulation from a distant ultrasound source and static culture using mineralisation staining, biomarker analysis, and histological protocols.

An *in vitro* culture of murine preosteoblasts (MC3T3-E1) confirmed the osteoconductivity of the rough titanium implant surface as a prelude to the *ex vivo* study: cell viability (>95%), cell proliferation and cell distribution was maintained for 28 days, and extensive networks of extracellular matrix formations were observed across the entire surface. The material choice and roughness (Ra = 75 µm) of this surface were both reflective of those used in contemporary cementless joint replacement implants.

In the *ex vivo* culture, calcium mineralisation on the active osseointegrative implant surfaces on the seventh and 14th days of treatment were highly elevated compared to the distant ultrasound specimens (some mineralisation) and the passive controls (near-zero mineralisation). SEM revealed extensive formations of irregular woven bone on the surface of the active osseointegrative implant by the 21-day treatment endpoint, and new growth emanating from bone fragments had begun to integrate with the surface. Bone formations were less expansive in distant ultrasound specimens and only small deposits were seen in the passive controls. Ongrown tissue on the active osseointegrative implant at the 21-day endpoint was also more viable than the passive controls. Histological and immunohistochemical analyses showed more active osteoid seams at the implant-bone interface in the active osseointegrative implant specimens compared to the specimens in the other conditions. Expression of osteopontin, a protein with a well-documented role in bone remodelling and cell adhesive interactions (Giachelli and Steitz, 2000; Icer and Gezmen-Karadag, 2018), was also elevated in osteocytes at the bone-implant interface in both the active osseointegrative implant (A; 2.5x greater, *p* < 0.005) and distant ultrasound specimens (B; 2x greater, *p* < 0.05). This finding implicates osteocyte-promoted cell attachment and new bone matrix deposition as underlying mechanisms for the increased ongrowth observed, with evidence that ultrasound still has some effect through 5 mm of bone, albeit less than direct stimulation from the active osseointegrative implant.

There was evidence of bone remodelling in the bulk trabecular structure of all specimens: remodelling zones exhibiting both osteoblasts and osteoclasts indicative of normal bone formation and resorption were observed during the histological and immunohistochemical analyses. Histological differences in the bulk trabecular structure of each specimen were less evident. Levels of alkaline phosphatase–an enzyme involved at the initiation of the mineralisation process (Golub and Boesze-Battaglia, 2007)–in the active osseointegrative implant specimens were lower than all the other samples on day 7, which may indicate a more rapid bone ossification process compared to the other samples. Levels of ALP expression decreased after the first week of culture, which was expected as ALP is an early biomarker, and no difference was detected between treatment groups. Calcium ion concentrations in the specimens treated by the active osseointegrative implant were elevated above the media baseline and the other specimens at every timepoint.

Before this study, *ex vivo* models had been used to study osseointegration of a passive orthopaedic implant, both in static (Zankovic et al., 2021) and dynamic loading conditions (Dua et al., 2021). In those studies, cell migration onto titanium implant surfaces had occurred within 4 weeks of culture (Dua et al., 2021; Zankovic et al., 2021), and immature crystallite-like structures had formed by the seventh week (Dua et al., 2021). Those findings were consistent with this research: cells had migrated onto the titanium implant surface within 3 weeks of passive culture but had not deposited mineralised tissue. *In vitro* models have previously been applied to study the isolated subprocesses of osseointegration: ultrasound stimulation promotes osteoblast differentiation (Nagasaki et al., 2015; Feng et al., 2019; Ambattu et al., 2022), maturation (Wang et al., 2014; Cao et al., 2017; Veronick et al., 2018), and proliferation (Wu et al., 2015), whilst electrical stimulation has been shown to promote osteoblast differentiation (Gittens et al., 2013), proliferation (Isaacson et al., 2011; Bodhak et al., 2012), and viability (Pettersen et al., 2021). With expansive mineralised structures within 3 weeks of treatment, this *ex vivo* model elevates evidence of the accelerated osteogenic effects of active osseointegrative treatment from cell level to tissue level.

Collectively, these results suggest that early osseointegration at the bone-implant interface (contact and distance osteogenesis) is enhanced by active stimulation. The distant ultrasound treatment demonstrated weaker enhancements in early osseointegration at the bone-implant interface but did not exhibit any adverse effects in the periprosthetic bone. Both treatment protocols used a single set of parameters, which were informed by literature, however their optimisation will require further work. Furthermore, the biomarkers investigated in this study, osteopontin and alkaline phosphatase, are early biomarkers, and the osteogenic effects of active treatment beyond 3 weeks remain unclear and may dissipate over time (Dergin et al., 2013). Such effects should be investigated in longer-term *ex vivo* cultures or *in vivo* animal models, with multiple timepoints and the inclusion of later-stage biomarkers, particularly osteocalcin. An *in vivo* model would also encompass the responses of other systems, which are known to affect osseointegration but whose interactions are not captured *ex vivo*. The increased electroactivity may have adverse effects in periprosthetic bone if pH levels are not regulated *in vivo*, but direct current electrical stimulation systems, which induce microenvironmental changes in pH with currents below 60 μA, have been deemed safe for clinical use (Nicksic et al., 2022). In a clinical embodiment, the requisite signals for the active osseointegrative implant can also be generated using lead-free transducers (Hall et al., 2023), with components otherwise hermetically sealed. The effects of treatment amidst clot formation (haemostasis) (Shiu et al., 2014) and the restoration of blood supply (angiogenesis) (Grosso et al., 2017), which are both orchestrated by the vascular system, are unknown but not expected to predominate the fundamental osteogenic response. Previously, a combination of stimuli has been required to promote a synergistically enhanced osteogenic-angiogenic response around an osseointegrative implant (Geng et al., 2020). This preclinical evaluation was conducted on healthy, skeletally immature porcine tissue, whilst osseointegrative implants are characteristically implanted into elderly patients, whose metabolisms, bone turnover, and mechanotransductive responses are all lower (Boskey and Coleman, 2010). Reduced osseointegration in elderly human tissue would therefore be expected in both passive and active conditions, though the relative effect on the response to treatment remains unclear. Further validation of treatment effects in this population could be obtained by repeating this evaluation with tissue sourced from arthroplasty procedures, such as femoral heads removed during total hip arthroplasty. Despite these limitations, this *ex vivo* model provides a platform for both optimising and understanding the early osteogenic effects of treatment as a cost-effective, 3Rs-compatible precursor to animal trials.

## 5 Conclusion

An innovative concept for active osseointegration was introduced and evaluated in an *ex vivo* bone model. Expansive extracellular structures with high cell viability and more than ten times higher mineralisation were observed on the surface of the active osseointegrative implant surface within 3 weeks of treatment, and bone tissue had begun to integrate. Contemporary cementless implant surfaces were insufficient to achieve rapid osseointegration in the passive condition: while cells had proliferated onto titanium implant surfaces within 3-4 weeks, there was little mineralisation. These early results therefore indicate an acceleration in the early phases of osseointegration with active treatment. The *ex vivo* model provides a platform for optimising and understanding active treatment under tightly controlled conditions at a tissue level as a cost-effective, low-ethical-impact alternative to *in vivo* animal trials.

## Data Availability

The original contributions presented in the study are included in the article/Supplementary Material, further inquiries can be directed to the corresponding author.
