# Supplementary material for: Active osseointegration in an ex vivo porcine bone model
Source: Front Bioeng Biotechnol. 2024 Mar 22;12:1360669. doi: 10.3389/fbioe.2024.1360669 (PMC10995341; doi:10.3389/fbioe.2024.1360669)
Supplement: Supplementary file 1 [file DataSheet1.pdf]

Active Osseointegration in an Ex Vivo Porcine Bone Model

Thomas A. G. Hall[1], Konstantinos Theodoridis[1], Nupur Kohli[1], Frederic Cegla[2], Richard J. van Arkel[1]

[1]Biomechanics Group, Department of Mechanical Engineering, Imperial College London, London, UK

[2]Non-Destructive Evaluation Group, Department of Mechanical Engineering, Imperial College London, London, UK

Supplementary Material

Histological analysis was conducted on all treatment and control conditions with bone sectioning in the plane perpendicular to the bone-implant interface in a sagittal-like plane (Fig. 5.B1). A series of images characterising the biological response of the bone tissue in response to each condition are provided as supplementary material (Figs. S2-S9).

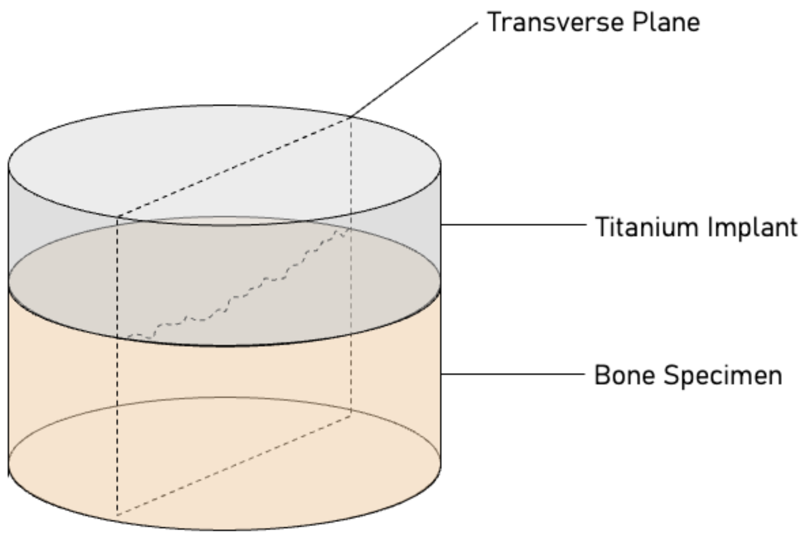

Figure S1. Bone specimens were sliced in the plane perpendicular to the bone-implant interface.

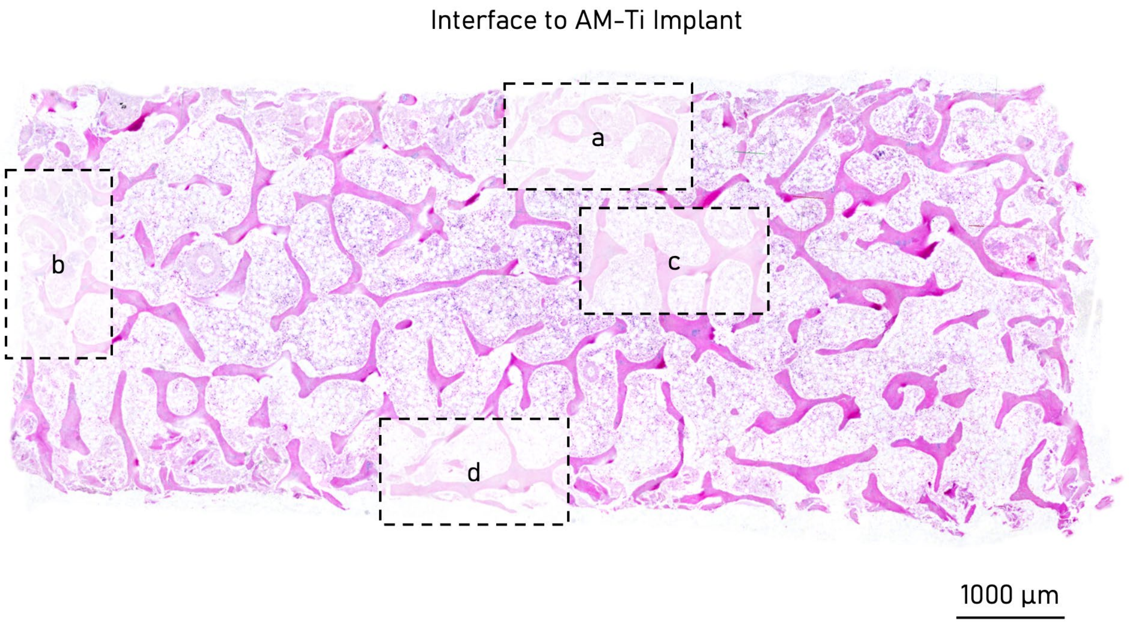

Figure S2. Macroscopic histological staining (H&E) of cancellous bone after 21 days of active osseointegrative implant treatment. Three regions of interest are identified for more detailed analysis: (a) tissue in apposition to the actively stimulated titanium surface; (b) wound tissue adjacent to the open surface of the sample; (c) non-interfacial bulk tissue; and (d) tissue adjacent to the polymer base of the bioreactor.

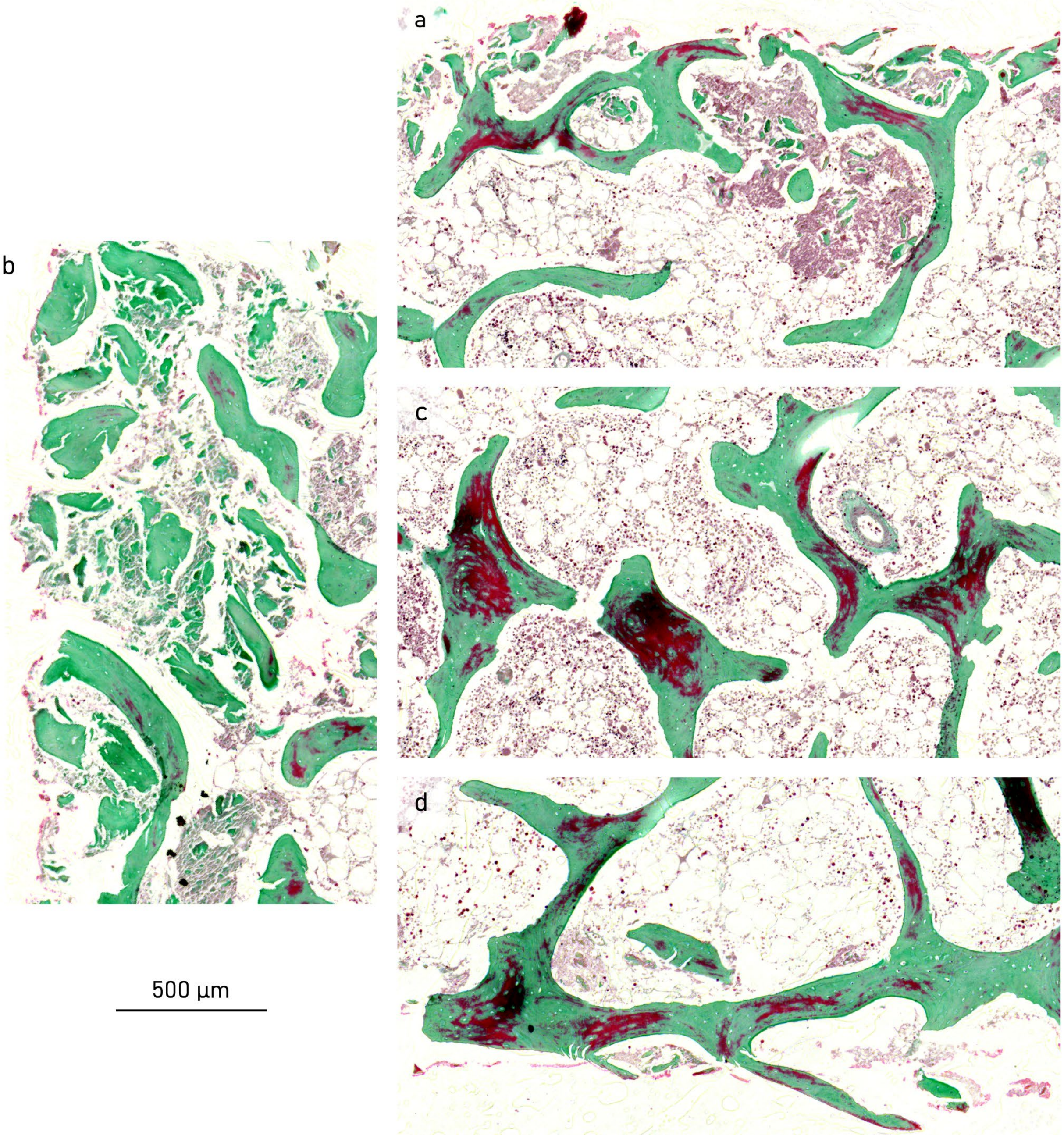

Figure S3. Masson-Goldner trichrome staining indicating osteoid seams and new bone matrix (both ruby red) after 21 days of active osseointegrative implant treatment (Treatment A) in representative regions of interest: (a) osteoid seams and new bone matrix are observed in direct apposition to the implant surface indicative of bone remodelling, with small bone fragments and fibrous tissue of haematopoietic origin in the bone marrow spaces; (b) less new bone matrix and osteoid seams are observed at the surface open to the media amidst debris from the original surgery; (c) new bone matrix is observed in the bulk tissue indicative of recent bone turnover; and (d) new bone matrix is observed on the opposite side of the implant without the fibrous tissue formation exhibited in apposition to the implant.

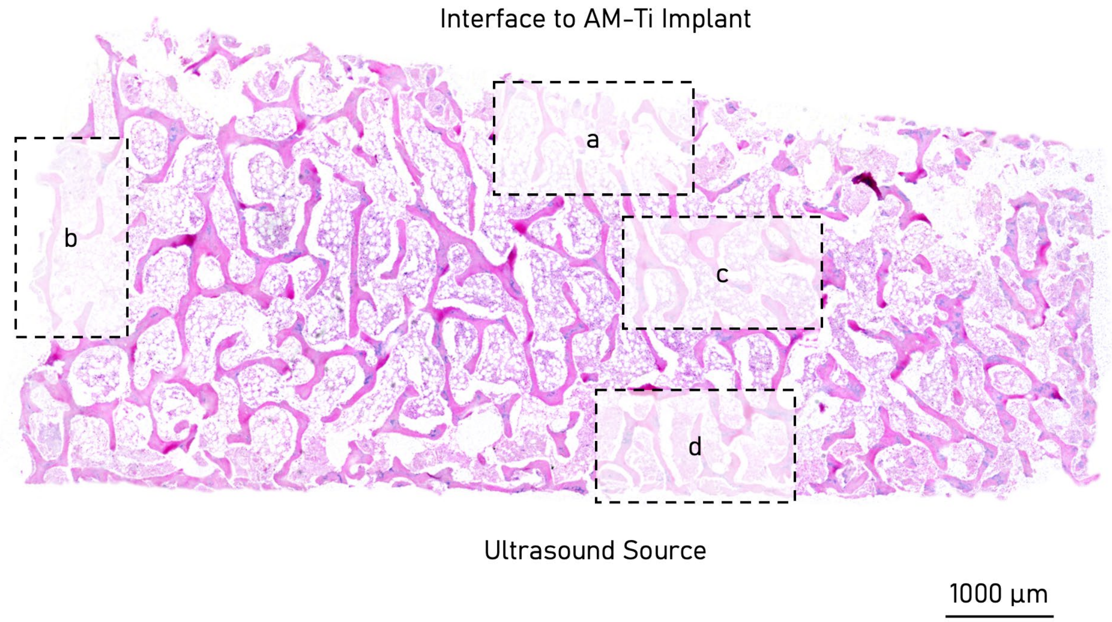

Figure S4. Macroscopic histological staining (H&E) of cancellous bone after 21 days of distant ultrasound treatment (Treatment B); tissue was sectioned in a sagittal-like plane. Three regions of interest are identified for more detailed analysis: (a) tissue adjacent to the titanium surface; (b) wound tissue adjacent to the open surface of the sample; (c) non-interfacial bulk tissue; and (d) tissue adjacent to the polymer base of the bioreactor where the ultrasonic treatment was applied.

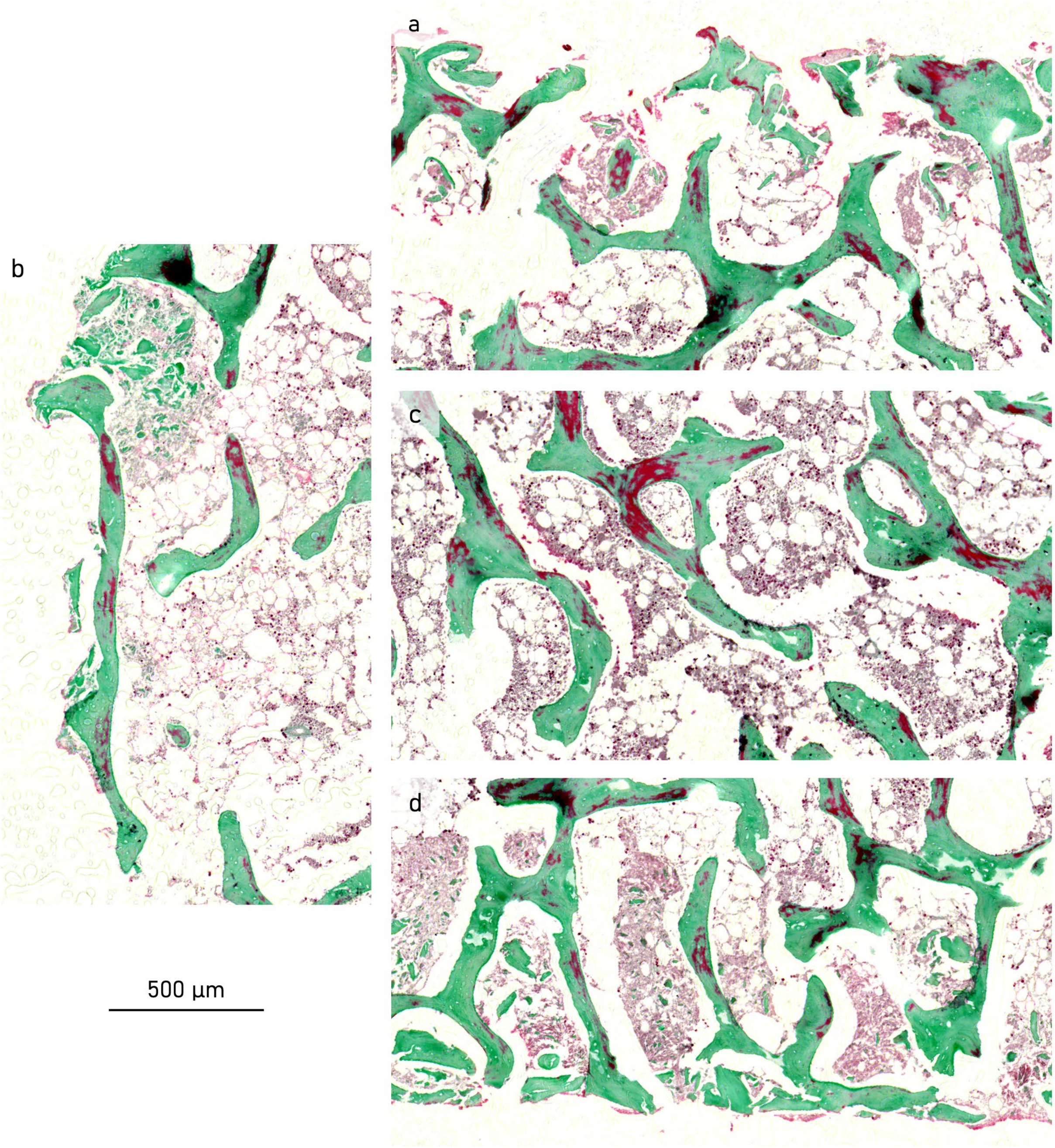

Figure S5. Masson-Goldner trichrome staining indicating osteoid seams and new bone matrix (both ruby red) after 21 days of distant ultrasound treatment (Treatment B) in representative regions of interest: (a) osteoid seams and new bone matrix were observed in direct apposition to the implant surface indicative of bone remodelling; (b) cutting debris from the original surgery with less new bone matrix and osteoid seams were seen at the surface open to the media; (c) new bone matrix was observed in the bulk tissue indicative of recent bone turnover; and (d) cutting debris and some evidence of active bone remodelling were seen in the tissue adjacent to the polymer base of the bioreactor where the ultrasonic treatment was applied.

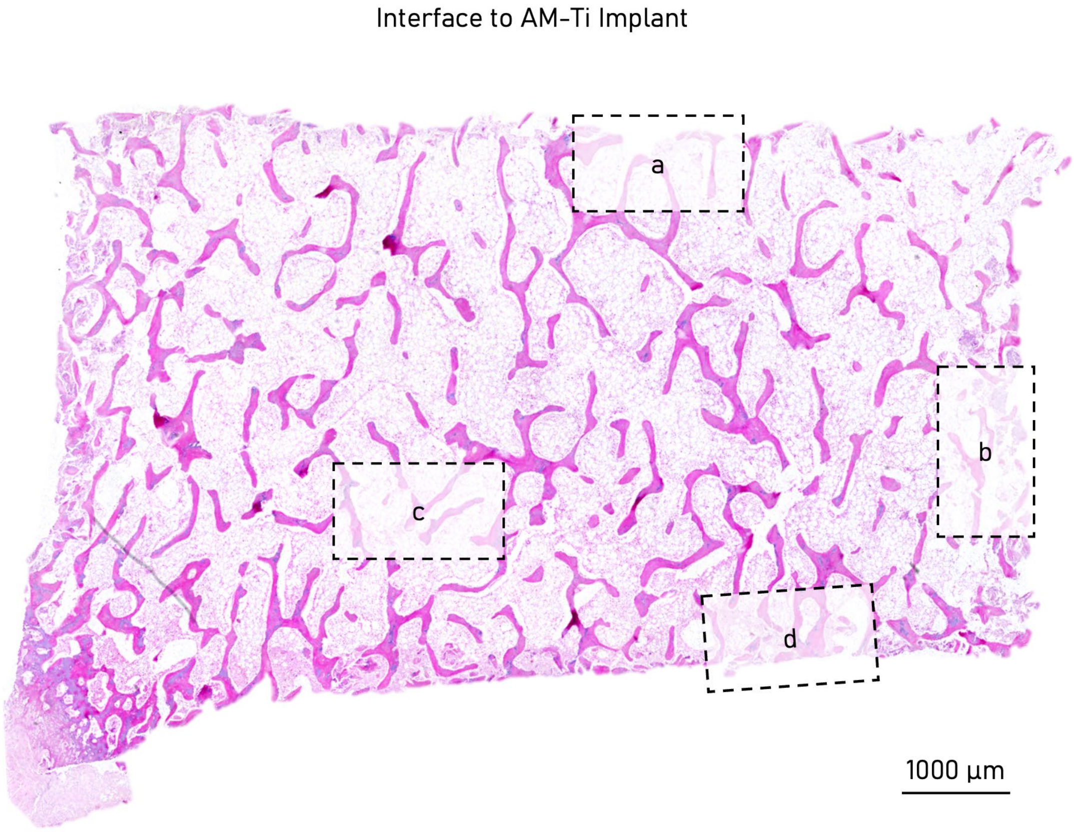

Figure S6. Macroscopic histological staining (H&E) of cancellous bone after 22-days for the passive implant control. Three regions of interest are identified for more detailed analysis: (a) tissue in apposition to the titanium surface; (b) wound tissue at the open surface of the sample; (c) non-interfacial bulk tissue; and (d) tissue adjacent to the polymer base of the bioreactor. A small amount of physeal tissue (growth plate) was extracted during the original resection of this specimen (bottom left), from which new tissue growth was emanating.

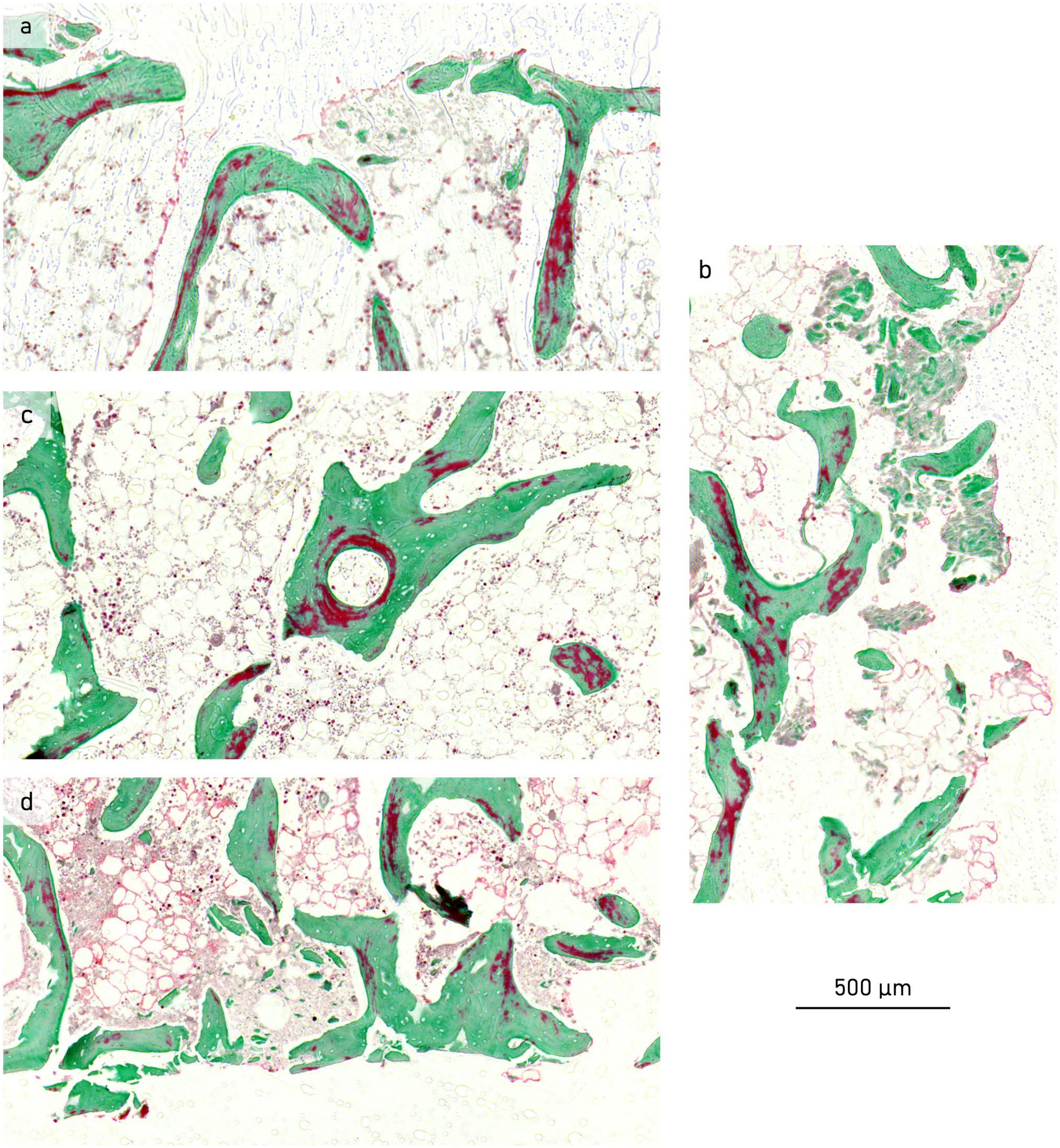

Figure S7. Masson-Goldner trichrome staining indicating osteoid seams and new bone matrix (both ruby red) for the passive implant control bone culture in representative regions of interest: (a) osteoid seams and new bone matrix were observed within the periprosthetic tissue but not in direct apposition to the implant; (b) cutting debris and some evidence of remodelling were seen at the surface open to the media; (c) new bone matrix was observed in the bulk tissue indicative of recent bone turnover; and (d) less evidence of new bone matrix was observed at the surface opposite the implant.

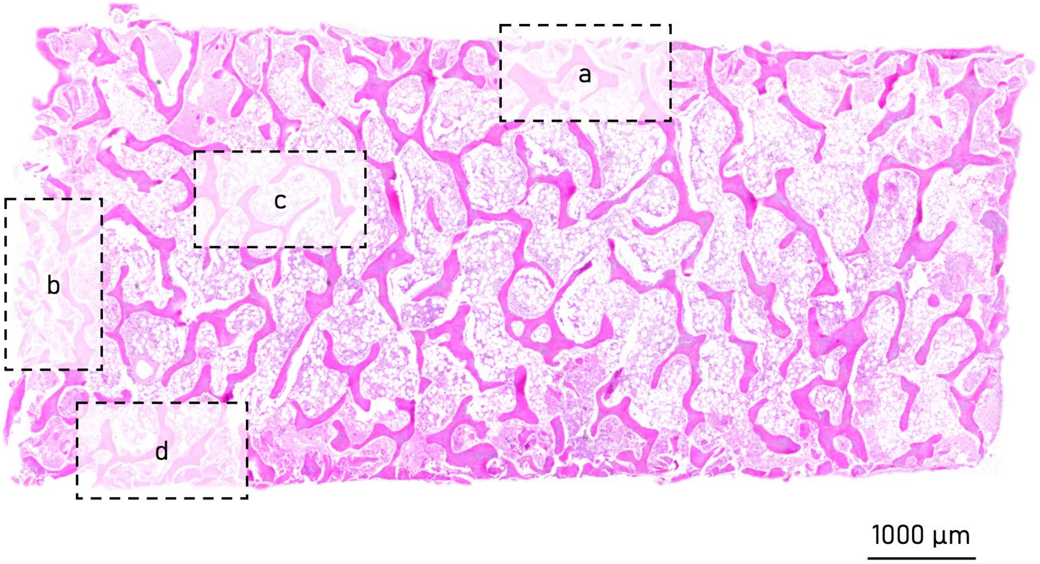

Figure S8. Macroscopic histological staining (H&E) of cancellous bone after 22-day bone-only culture. Three regions of interest are identified for more detailed analysis: (a) tissue that was cut using an oscillating saw and was open to the media; (b) tissue that was cut using a holesaw and was open to the media; (c) non-interfacial bulk tissue; and (d) tissue adjacent to the polymer base of the bioreactor.

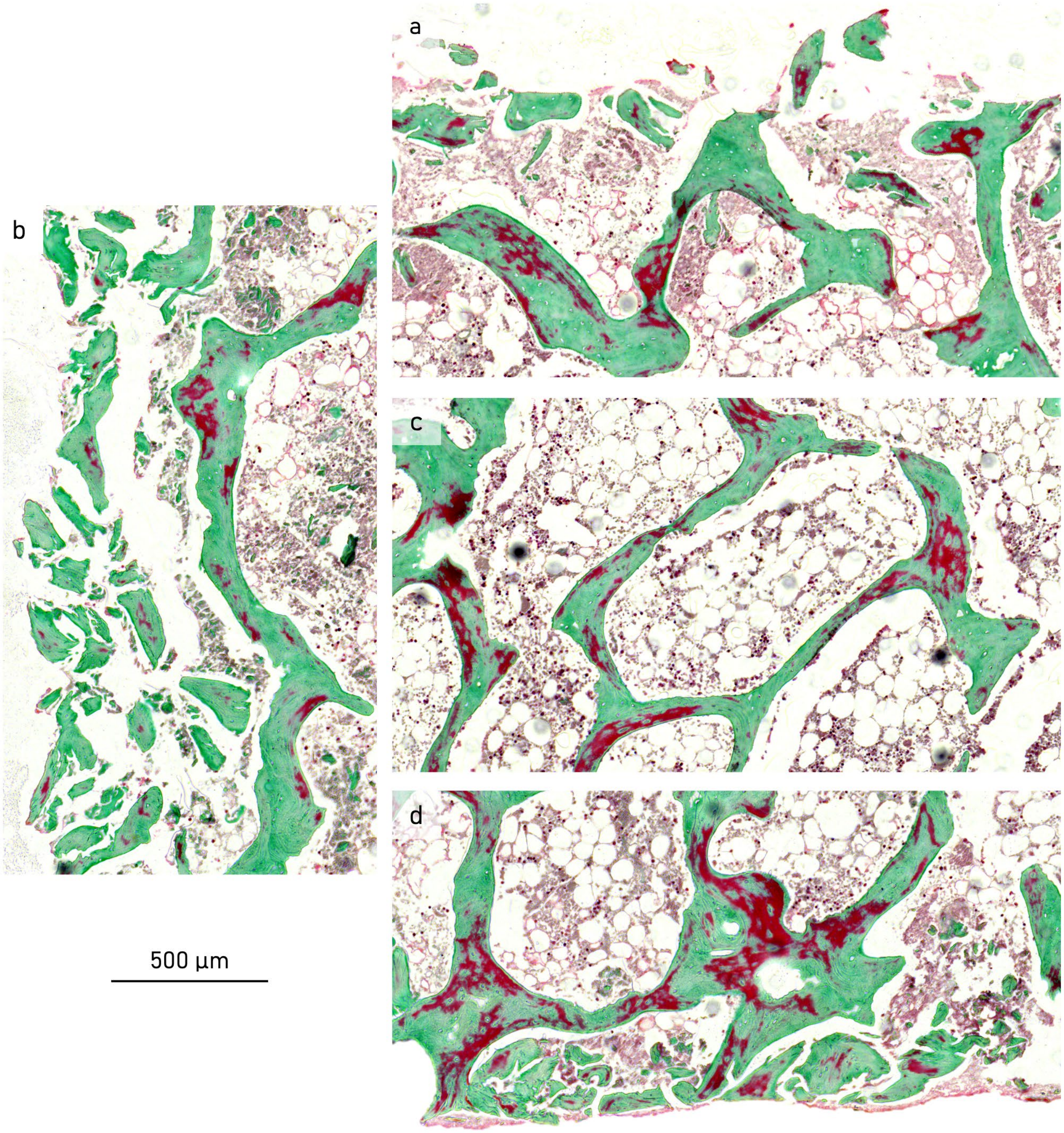

Figure S9. Masson-Goldner trichrome staining indicating osteoid seams and new bone matrix (both ruby red) after bone-only culture in representative regions of interest: (a) osteoid seams and new bone matrix were observed at the open face of the oscillating-saw cut bone surface exposed to media; (b) little evidence of remodelling was seen amidst the cutting debris of the hole-saw cut side of the cylinder; (c) new bone matrix was observed in the bulk tissue indicative of recent bone turnover; and (d) less evidence of new bone matrix was observed at the bone-surface adjacent to the polymer bioreactor base.
